# Comparative Genomics Insights into a Novel Biocontrol Agent *Paenibacillus peoriae* Strain ZF390 against Bacterial Soft Rot

**DOI:** 10.3390/biology11081172

**Published:** 2022-08-04

**Authors:** Yurong Zhao, Xuewen Xie, Junhui Li, Yanxia Shi, Ali Chai, Tengfei Fan, Baoju Li, Lei Li

**Affiliations:** Institute of Vegetables and Flowers, Chinese Academy of Agricultural Sciences, Beijing 100081, China

**Keywords:** cucumber soft rot, *Paenibacillus peoriae*, comparative genome analysis, PGPR, antagonistic activity, biocontrol agents, biocontrol mechanisms

## Abstract

**Simple Summary:**

Bacterial soft rot, attributed to *Pectobacterium brasiliense* infection, has caused destructive impacts and colossal economic losses to China’s agricultural industry. Chemical control, which was ubiquitously used, cannot manage this disease as expected, so biocontrol has been followed with interest to date. In this study, we found a *Paenibacillus peoriae* strain ZF390 that had a potent control efficiency over cucumber plants against *Pectobacterium brasiliense*, and the comparative genomic analysis revealed biocontrol mechanisms might be involved in the strain ZF390.

**Abstract:**

Bacterial soft rot, caused by *Pectobacterium brasiliense*, can infect several economically important horticultural crops. However, the management strategies available to control this disease are limited. Plant-growth-promoting rhizobacteria (PGPR) have been considered to be promising biocontrol agents. With the aim of obtaining a strain suitable for agricultural applications, 161 strains were isolated from the rhizosphere soil of healthy cucumber plants and screened through plate bioassays and greenhouse tests. *Paenibacillus peoriae* ZF390 exhibited an eminent control effect against soft rot disease and a broad antagonistic activity spectrum in vitro. Moreover, ZF390 showed good activities of cellulase, protease, and phosphatase and a tolerance of heavy metal. Whole-genome sequencing was performed and annotated to explore the underlying biocontrol mechanisms. Strain ZF390 consists of one 6,193,667 bp circular chromosome and three plasmids. Comparative genome analysis revealed that ZF390 involves ten gene clusters responsible for secondary metabolite antibiotic synthesis, matching its excellent biocontrol activity. Plenty of genes related to plant growth promotion, biofilm formation, and induced systemic resistance were mined to reveal the biocontrol mechanisms that might consist in strain ZF390. Overall, these findings suggest that strain ZF390 could be a potential biocontrol agent in bacterial-soft-rot management, as well as a source of antimicrobial mechanisms for further exploitation.

## 1. Introduction

*Pectobacterium brasiliense* (*Pbr*), considered one of the most virulent species among the *Pectobacteriaceae*, has a wide host range and causes soft rot on miscellaneous growing plants or harvested crops, including important food crops and ornamentals [1,2]. In addition to blackleg disease on potatoes, *Pbr* causes a devastating soft rot of cucumber in China, with an incidence that varies from 15 to 50% in different fields, causing 20 to 30% yield losses in five provinces during 2014–2015 [2,3,4]. The deficiency of the effective resistance present in commercial cultivars and practical chemical control agents makes the management of soft rot *Pectobacteriaceae* (SRP) challenging.

In recent decades, biocontrol strategies, which are more novel, cost-effective, and environmentally and human-safe than other control strategies, have become promising tools to reduce losses of vegetables [2,5]. Similar to *Bacillus*, the *Paenibacillus* species can be applied in agriculture as phytostimulators, biofertilizers, and bioagents. *Paenibacillus* strains have exhibited their biocontrol properties, including plant colonization, pathogen inhibition, plant-growth promotion abilities, and the activation of induced systemic resistance [6,7]. Previous studies revealed potential *Paenibacillus* biocontrol agents to protect plants from pathogens such as *Phytophthora tropicalis*, *P. parasitica*, *Fusarium oxysporum*, and *Xanthomonas oryzae* [8,9,10,11]. Nonetheless, the available resources and genetically explored genome sequences of *Paenibacillus* to date are far fewer than those of *Bacillus* [12,13]. Thus, *Paenibacillus* has attracted considerable attention, owing to its potential antagonistic microbial properties and the relative scarcity of reported biocontrol strains [14,15].

Furthermore, motility toward plant roots and biofilm formation on the root surface are crucial for the colonization of plant roots and biocontrol efficacy against plant pathogens [16,17,18]. The production of active substances is an important indicator for assessing the biocontrol efficacy of a beneficial strain [19,20], including lipopeptide antibiotics, bacteriocins, and antibacterial proteins [21]. In addition, key genes are responsible for the production of antibacterial agents and the volatile organic compounds, indole acetic acid synthesis, siderophore secretion, quorum sensing, and biosynthesis of two-component systems [22]. Currently, comparative genomics has been recognized as an important tool for identifying and understanding major biological control mechanisms and key functional genes in related organisms, providing useful inferences for their related biocontrol mechanisms [22,23].

In this study, *Paenibacillus peoriae* ZF390 was obtained from the healthy rhizosphere soil of cucumber plantations with a high incidence of cucumber soft rot [24]. Phylogenetic, ANI, and in silico DDH analyses were performed to define the taxonomic position of ZF390 and its relationship with other *P. peoriae* strains. Through comparative genomics and functional gene analysis, it was found that strain ZF390 has 10 secondary metabolite synthesis gene clusters and key genes related to various mechanisms, such as plant growth promotion, disease prevention, and environmental adaptation. This study provides technical support and a theoretical basis for the study of the biocontrol mechanism of strain ZF390.

## 2. Materials and Methods

### 2.1. Bacterial Isolation, Growth Conditions, Antagonism Assays, and Biocontrol Assays

Strain ZF390 was derived from the healthy rhizosphere soil of cucumber plantations in Harbin, China, in August 2019. According to a previous study [24], 10 g of the soil sample was subjected to 10,000-fold dilution with sterile water, plated on LB plates, and incubated at 28 °C for 48 h. *Bacillus*-like and *Paenibacillus*-like strains were retained for further investigations. All the strains were purified on LB plates and cultivated in LB broth at 28 °C with shaking for 24 h.

The antagonistic activity of selected microorganisms against *Pbr* was estimated through plate bioassays. The suspensions of all the isolated strains were diluted to an OD_600_ = 0.6 with sterile LB broth. Then, 5 μL of the suspensions were inoculated in the center of PDA plates and cultivated for 24 h, respectively. A total of 3 mL of chloroform was added to each upside-down dish for fumigation to inactivate the bacteria colony. Subsequently, 100 μL of the suspension of *Pbr* was equably added into 5 mL WA medium, and the mixture was poured on top of the PDA plates. If the isolated strain possessed the ability to against *Pbr*, the plate would change from white to transparent. The suppressive activity test of strain ZF390 against different kinds of phytopathogenic bacteria was similar to the aforesaid method. The transparent aperture appeared on the plate if the strain ZF390 had the capacity to defend against the pathogen. In addition, the inhibitory effect of strain ZF390 against various plant pathogenic fungi was assessed through plate assays. The fungus dishes with diameters of 5 mm were inoculated on the center of PDA plates, and 5 μL of ZF390 suspension was diluted to an OD_600_ = 0.6 with sterile LB broth that was inoculated at the spots 3.5 cm away from the dishes. Fungal hyphae were inhibited after the plates were cultured at 28 °C for 7–10 d if strain ZF390 could defend against the phytopathogenic fungi.

The control efficacy of antagonistic strains against cucumber bacterial soft rot disease was assessed by greenhouse experiments with potted cucumbers. Each treatment contained five healthy cucumber seedings sprayed with 200 mL suspension of *Pbr* diluted to an OD_600_ = 0.8 with sterile NB broth. The greenhouse was kept at 28 °C and 75% relative humidity for 24 h. Whereafter, 3 mL antagonistic strain suspension was subjected to 100-fold dilution with sterile water and spray inoculated on the cucumber seedings. The chemical pesticide group was treated with 300 mL of 3% Zhongshengmycin wettable powder 650-fold dilution, and the 300 mL sterile water served as a control group. The disease grade was divided into five grades: absence of spots represented grade 0; grade 1 was classified as a spot area less than 5% of leaf area; the proportion of spot area from 6% to 25% was grade 2; the proportion of spot area from 26% to 50% was grade 3; the proportion from 51% to 75% was grade 4; and the spot area larger than 76% was grade 5. Disease index = 100 × ∑ (the number of leaves in certain disease grade × relative disease grade)/(total number of leaves × the highest grade of disease). Control effect (%) = 100 × (disease index of control group—disease index of treatment group)/disease index of control group.

In addition, the activities of cellulase, protease, and phosphatase were measured using previously reported methods [25,26]. If cellulase was produced, the CMC agar plate would change from red to yellow after staining with 0.1% (*w*/*v*) Congo red solution for 30 min and washing with 1 M NaCl solution for 15 min. While, if protease and phosphatase were produced, haloes in plates became visible without further treatment. The heavy-metal tolerance of strain ZF390 was analyzed using a standard 10-fold dilution plating assay. Different concentrations of ZF390 suspension were inoculated on the LB plates supplemented with 0.4 mM CdCl_2_·2^1^/_2_H_2_O, 1 mM CoCl_2_·6H_2_O, 3 mM MnSO_4_·H_2_O, 5 mM PbCO_3_, and 3 mM ZnCl_2_, respectively. Additionally, the plates were incubated at 28 °C for 48 h. All of the above experiments were repeated three times.

### 2.2. Characteristics, Microscopic Analysis and Genomic DNA Extraction

The growth characterization and physiological and biochemical characteristics were determined on the basis of reported approaches [27,28]. The strain morphologies were observed by transmission electron microscopy (TEM) and scanning electron microscopy (SEM). Genomic DNA was extracted from the cultured ZF390 cells using the TIANamp Bacteria DNA Kit (TIANGEN BIOTECH, Beijing, China).

### 2.3. Genome Sequencing and Annotation

According to the manufacturer’s protocol, the whole genome of strain ZF390 was subjected to single-molecule real-time (SMRT) sequencing on the Pacific Biosciences Sequel (Pacific Biosciences, Menlo Park, CA, USA) platform using a library prepared with the SMRTbell Template Prep Kit 1.0 (Pacific Biosciences, Menlo Park, CA, USA). The reads were assembled de novo using SMRT LINK v5.0 (Pacific Biosciences, Menlo Park, CA, USA) after qualitative filtration. The circular genome visualization was generated using CGView [29]. Coding genes of the sequenced genome were predicted by GeneMarkS software. tRNAscan-SE, rRNAmmer, and cmsearch were used to predict tRNA (transfer RNA), rRNA (ribosomal RNA), and snRNAs (small nuclear RNAs), respectively [30,31,32]. Functional gene annotation was carried out through multiple general databases, including nonredundant protein databases (NR) [33], the Gene Ontology database (GO) [34], the Kyoto Encyclopedia of Genes and Genomes (KEGG) [35], the Cluster of Orthologous Groups of proteins (COG) [36], the Transporter Classification Database (TCDB) [37], Pfam3 Swiss-Prot4 [38], and the Carbohydrate-Active enZTmes Database (CAZy) [39]. Furthermore, phiSpy was used to predict prophages, and clustered, regularly interspaced, short palindromic repeat sequences (CRISPR) were identified via CRISPRdigger 1.0 [40,41].

### 2.4. Phylogenetic Analysis and Genome Comparisons

The taxonomic position of strain ZF390 was determined using multilocus gene sequence analysis (MLSA) based on eight housekeeping genes (*gyrB*, *rpoD*, *rplA*, *atpD*, *holB*, *rho*, *dnaA* and *rpsA*). The gene sequences were aligned using MUSCLE and trimmed to remove ambiguously aligned regions (the final sequence lengthes were 9910 bp). Subsequently, the phylogenetic tree was constructed using the maximum likelihood method in MEGA 7.0 [42]. Other available gene sequences of the *Paenibacillus* strains to be used for phylogenetic tree construction were downloaded from the NCBI database. According to the phylogenetic analysis, four closely related *Paenibacillus* strains with released complete genome sequences, including *P. peoriae* HS311 (GenBank Accession No. CP011512.1), *P. polymyxa* HY96-2 (GenBank Accession No. CP025957.1), *P. polymyxa* SQR-21 (GenBank Accession No. CP006872.1), and *P. kribbensis* PS04 (GenBank Accession No. 041731.1), were selected for genome comparison with ZF390. Average nucleotide identities (ANI) and in silico DNA–DNA hybridization (*is*DDH) analyses were performed using the OrthoANIu algorithm and the Genome-to-Genome Distance Calculator (GGDC), respectively [43]. Moreover, the pairwise alignment of genomes was generated using the Mauve v2.3.1 (SourceForge, San Diego, CA, USA) and Venn diagrams were constructed using the R package [44].

### 2.5. Analysis of Gene Clusters for Biosynthesis of Secondary Metabolites

The secondary metabolite gene cluster prediction was performed using antiSMASH 5.0 on the authors’ Web servers using the default parameters [45]. Comparative analyses of secondary metabolite gene clusters identified that, among strain ZF390, *P. peoriae* HS311, *P. polymyxa* HY96-2, *P. polymyxa* SQR-21, and *P. kribbensis* PS04 were performed based on the Kyoto Encyclopedia of Genes and Genomes database (KEGG) [35] and the GenBank database.

### 2.6. Genome Mining for Genes Encoding Plant Beneficial Traits

The functional genes involved in plant-growth promotion or plant–bacterial interactions, such as the genes responsible for plant-growth promotion, biofilm formation, and induced systemic resistance, were searched in the NCBI and KEGG databases. The identities of different functional genes at the amino acid level were compared between strain ZF390, *P. peoriae* HS311, *P. polymyxa* HY96-2, *P. polymyxa* SQR-21, and *P. kribbensis* PS04 using BLAST (Basic Local Alignment Search Tool).

### 2.7. Statistical Analysis

All data were analyzed using the analysis of variance in SPSS v24.0 (IBM, Armonk, NY, USA). The significant differences between means were compared by using the LSD test (Fisher’s protected least significant differences test) at *p* = 0.05. A *p* value < 0.05 was considered significant.

## 3. Results

### 3.1. Biocontrol Effect of Strain ZF390 against Pbr

**Figure 1:**
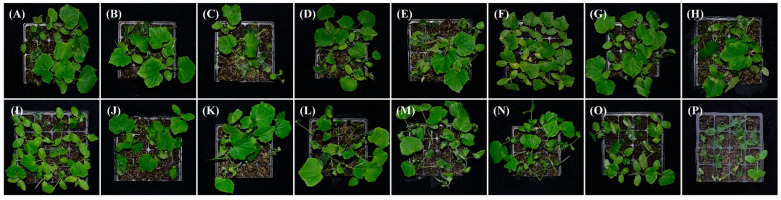
Control efficiency of antagonistic strains against bacterial soft rot disease on potted cucumbers. (**A**): ZF115; (**B**): ZF119; (**C**): ZF185; (**D**): ZF194; (**E**): ZF278; (**F**): ZF390; (**G**): ZF402; (**H**): ZF405; (**I**): ZF428; (**J**): ZF429; (**K**): ZF436; (**L**): ZF448; (**M**): ZF450; (**N**): ZF453; (**O**): Zhongshengmycin; and (**P**): Control of sterilized water.

To identify good biocontrol agents against cucumber soft rot disease caused by *Pbr*, 161 bacterial strains were segregated from the rhizosphere of cucumber plants. There were 14 strains that showed antagonistic activity against *Pbr* through plate bioassays (Supplementary Figure S1). The largest transparent halo was generated around the colony of strain ZF428, while only a small ring was produced around the colony of strain ZF390 (Supplementary Figure S1). The control effects of these 14 strains against *Pbr* were performed by greenhouse experiments on cucumber seedlings. Among these bacteria, the strain ZF390 indicated superb antagonistic activity in greenhouse tests, exhibiting the highest control efficiency of 77.47%, which was much higher than the efficacy of zhongshengmycin (47.89%) [46], which is a ubiquitous aminoglycoside antibiotic against bacterial phytopathogens (Table 1 and Figure 1).

**Figure 2:**
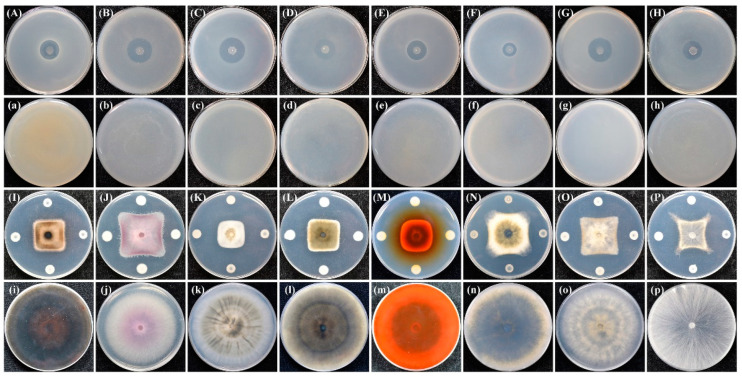
Antagonistic assay of *Paenibacillus peoriae* ZF390 against eight pathogenic bacteria and eight pathogenic fungi. (**A**) and (**a**): *Pectobacterium brasiliense*; (**B**) and (**b**): *Xanthomonas campestris* pv. *campestris*; (**C**) and (**c**): *Pseudomonas syringae* pv. *tomato*; (**D**) and (**d**): *Clavibacter michiganensis* subsp. *michiganensis*; (**E**) and (**e**): *Pseudomonas syringae* pv. *lachrymans*; (**F**) and (**f**): *Clavibacter michiganensis* subsp. *sepedonicum*; (**G**) and (**g**): *Agrobacterium vitis*; (**H**) and (**h**): *Acidovorax citrulli*; (**I**) and (**i**): *Corynespora cassiicola*; (**J**) and (**j**): *Fusarium oxysporum*; (**K**) and (**k**): *Colletotrichum capsici*; (**L**) and (**l**): *Phytophthora capsici*; (**M**) and (**m**): *Stemphylium solani*; (**N**) and (**n**): *Ascochyta citrulline*; (**O**) and (**o**): *Botrytis cinerea*; and (**P**) and (**p**): *Rhizoctonia solani*. Uppercase letters indicated the treatment; lowercase letters indicated the negative control.

The strain ZF390 also demonstrated prominent antagonistic activities against various plant bacterial and fungal pathogens, including *Pbr*, *X. campestris* pv. *Campestris*, *Pseudomonas syringae* pv. *Tomato*, *Clavibacter michiganensis* subsp. *Michiganensis*, *P. syringae* pv. *Lachrymans*, *C. michiganensis* subsp. *Sepedonicum*, *Agrobacterium vitis*, *Acidovorax citrulli*, *Corynespora cassiicola*, *F. oxysporum*, *Colletotrichum capsici*, *P. capsici*, *Stemphylium solani*, *Ascochyta citrulline*, *Botrytis cinerea*, and *Rhizoctonia solani* in the plate bioassays (Figure 2). In addition, yellow and transparent halos were observed in extracellular enzyme assays, indicating that strain ZF390 produced cellulase, protease, and phosphatase (Supplementary Figure S2A–C), which might be one of its biocontrol mechanisms. Moreover, the strain ZF390 revealed varying degrees of tolerance to Cd^2+^, Co^2+^, Mn^2+^, Pb^2+^, and Zn^2+^ (Supplementary Figure S2D). Strain ZF390 exhibited a superior colonization ability in the rhizospheres of cucumbers cultivated in fermentable substrate after 15 days of inoculation (1.36 × 10^8^ CFU/g) (Supplementary Figure S3). In summary, due to the remarkable biocontrol traits of strain ZF390, it was selected for further research.

### 3.2. Organism Information

**Figure 3:**
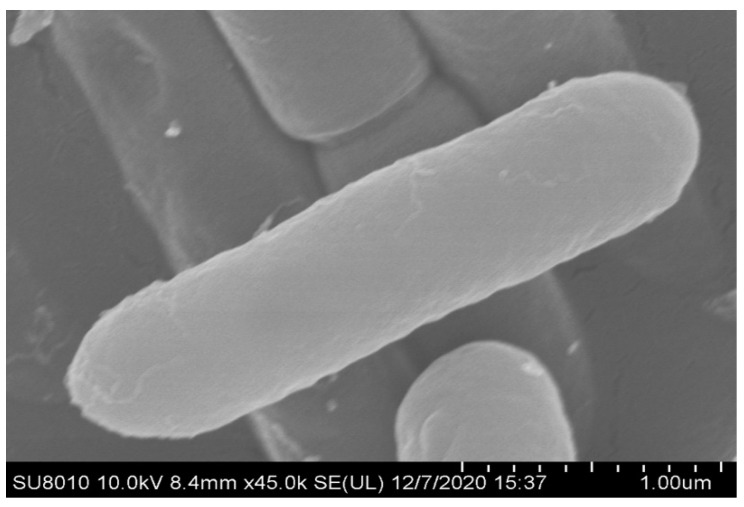
Image of ZF390 cells using scanning electron microscope.

Strain ZF390 was demonstrated to be a motile, Gram-positive, and facultative anaerobic bacterium belonging to the *Paenibacillaceae* family. Strain ZF390 grew readily on LB plates at 28 °C and produced light yellow sticky colonies with irregular margins after 36 h of incubation (Supplementary Figure S4A), and the strain produced rod-shaped cells with a length of 2–4 μm and a diameter of 0.5–1 μm (Figure 3 and Supplementary Figure S4B). As indicated in the physiological and biochemical assays, casein was hydrolyzed by strain ZF390, but arginine dihydrolase and indole were not produced, and the nitrate reduction reaction was negative. Interestingly, the capability of strain ZF390 to liquefy gelatin was negative, which was the complete opposite of that of *P. peoriae* KCTC 3763^T^ [28]. Acid was produced by ZF390 from the following compounds: glycerol, D-arabinose, L-arabinose, ribose, D-xylose, adonitol, methylxyloside, D-fructose, mannitol, methyl-D-mannoside, salicin, maltose, D-trehalose, gentiobiose, 2-keto-D-gluconate, 5-keto-D-gluconate, rhamnose, D-turanose, and N-acetylglucosamine, and its capacity of utilizing D-turanose was different from that of KCTC 3763^T^ (Supplementary Table S1).

### 3.3. General Genomic Features of P. peoriae ZF390

**Figure 4:**
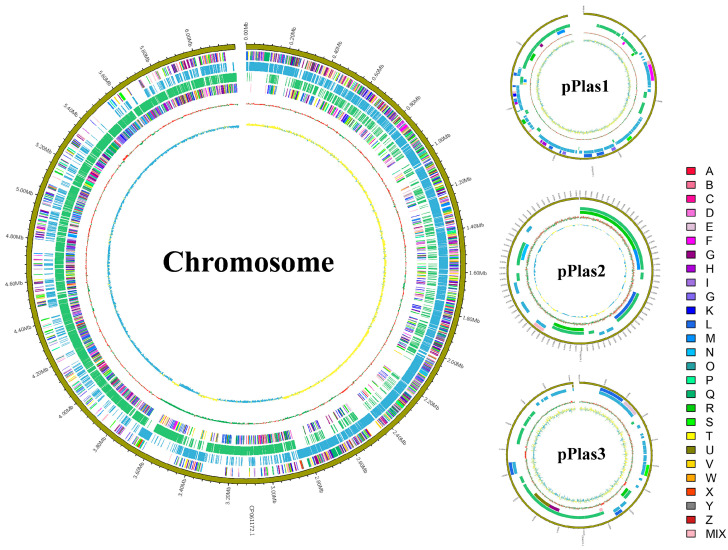
Graphical circular maps of the *Paenibacillus peoriae* ZF390 chromosome and plasmids pPlas1, pPlas2, and pPlas3 generated with CGview Server. From outside to center, rings 2 and 5 show protein-coding genes oriented in the forward (colored by COG categories) and reverse (colored by COG categories) directions, respectively. Rings 3 and 4 show genes on the forward and reverse strands. Ring 6 shows the GC content plot, and the innermost ring shows the GC skews, where yellow indicates positive values, and blue indicates negative values.

The complete genome of *P. peoriae* ZF390 was composed of a circular 6,193,667 bp chromosome with three additional plasmids, pPlas1 (137,220 bp), pPlas2 (16,869 bp), and pPlas3 (36,233 bp), and the average GC contents were 44.96%, 45.58%, 45.12%, and 41.66%, respectively (Supplementary Table S2). In total, 5890 open reading frames (ORFs) were forecasted in the genome of ZF390. In addition to 5574 protein-coding genes (CDSs), the genome constituted 145 RNA genes, containing 40 rRNA operons, 101 tRNA genes, 4 other RNAs, and 171 pseudogenes (Supplementary Table S3). In addition, the ZF390 genome included 17 genomic islands, 5 prophage regions, and 11 CRISPR-associated genes (Supplementary Table S2). A total of 4001 functional genes of the ZF390 genome were categorized using the Clusters of Orthologous Groups of proteins (COG) database. Most of the genes were related to carbohydrate transport and metabolism (505 genes), followed by transcription (482 genes), general function prediction only (451 genes), amino acid transport and metabolism (355 genes), and signal transduction mechanisms (304 genes). However, 439 genes were not predicted by the COG database, and their specific functions need to be further verified (Supplementary Table S4). The graphical circular genomic maps exhibiting the genome structure and functions of *P. peoriae* ZF390 are presented in Figure 4. Moreover, 5402 genes were annotated to 204 different KEGG pathway maps, the majority of which were associated with metabolic pathways, biosynthesis of secondary metabolites, ABC transporters, the biosynthesis of antibiotics, microbial metabolism in diverse environments, two-component systems, the biosynthesis of amino acids, and quorum sensing. In addition, 3754, 5645, 481, 3754, 2296, and 342 ORFs were compared to diverse pathways or domains using the GO, NR, TCDB, Pfam, Swiss-Prot, and CAZy databases (Supplementary Table S2).

### 3.4. Comparative Genomics Analysis of P. peoriae ZF390 with Other Paenibacillus Strains

**Figure 5:**
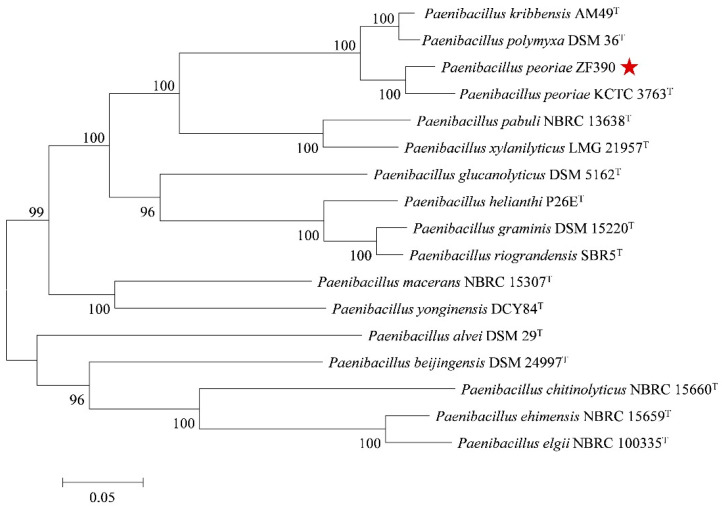
Phylogenetic tree highlighting the relative position of *P. peoriae* ZF390 among other *Paenibacillus* species. The phylogenetic tree was constructed based on eight housekeeping genes (*gyrB*, *rpoD*, *rplA*, *atpD*, *holB*, *rho*, *dnaA* and *rpsA*) according to the aligned gene sequences using the maximum likelihoods method in MEGA 7.0 software. Bootstrap values (1000 replicates) are shown at the branch points. The scale bar indicates 0.05 nucleotide substitution per nucleotide position. GenBank accession numbers associated with the housekeeping loci for all strains are presented in Supplementary Table S5. The red star marks the location of strain ZF390.

To comprehend the genetic relationship of *P. peoriae* ZF390 with other *Paenibacillus* species, sixteen publicly available complete genome sequences of *Paenibacillus*-type strains were selected to establish a phylogenetic tree based on eight housekeeping genes (*gyrB*, *rpoD*, *rplA*, *atpD*, *holB*, *rho*, *dnaA*, and *rpsA*) (Supplementary Table S5). As expected, all of the strains were prominently corroborated by bootstrap values. According to the genetic distance, strain ZF390 was closest to *P. peoriae* KCTC 3763^T^, followed by *P. polymyxa* DSM 36^T^ and *P. kribbensis* AM49^T^ (Figure 5). The phylogenetic analysis determined an accurate systematic position of ZF390 as *P. peoriae*.

ANI and *is*DDH are widely applied analyses that appraise the similarities between bacterial strains by comparing whole genomic sequence data. Pairwise compared strains with ANI values ≥ 96% and *is*DDH values ≥ 70% are typically considered the same species [47,48]. In this study, the ANI and *is*DDH values between strains ZF390 and HS311 were 96.61% and 71.80%, respectively (Supplementary Figure S5). Simultaneously, the ANI values were over 96%, and the *is*DDH values were over 70%, when comparing strain ZF390 with *P. polymyxa* strains ATCC 15970, CR1, E681, J and YC0573. Nevertheless, lower ANI and *is*DDH values were obtained when other *P. polymyxa* strains were used as reference genomes. These findings may suggest inaccurate identities of partial *P. polymyxa* strains. In addition, the ANI values that contrasted strain ZF390 with *P. kribbensis* strains AM49^T^ and PS04 were 87.46% and 87.41%, respectively, and the *is*DDH values were 33.70% and 33.80%, respectively. Altogether, these results revealed that *P. polymyxa* and *P. kribbensis* were in more appressed relationships with *P. peoriae* than other *Paenibacillus* species. Strain ZF390 was closely related to *P. peoriae* HS311 and could be assigned to the same taxonomic position (Supplementary Figure S5).

Genomic information was compared between *P. peoriae* ZF390 and four other accessible complete genomes of *Paenibacillus* strains (*P. peoriae* HS311, *P. polymyxa* SQR-21, *P. polymyxa* HY96-2 and *P. kribbensis* PS04) (Supplementary Table S3). The genome size of strain ZF390 (6.38 Mb) was conspicuously large compared to the other four strains, HS311 (6.22 Mb), SQR-21 (5.83 Mb), HY96-2 (5.75 Mb), and PS04 (5.74 Mb). These results also illustrate a maximum number of ORFs and CDSs in the ZF390 genome. Nevertheless, the order of average GC content of these five selected strains was antipodal with the order of genome size results; strain ZF390 had the lowest GC content (44.99%), under 45%. Furthermore, *P. peoriae* ZF390 and HS311 comprised three plasmids and one plasmid, respectively, and the other three *Paenibacillus* strains merely contained a single chromosome without a plasmid (Supplementary Table S3).

**Figure 6:**
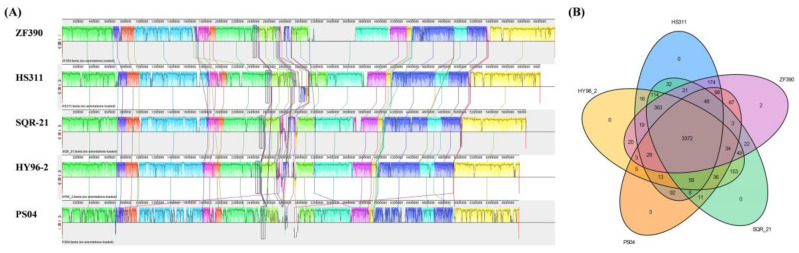
Comparison of *P. peoriae* ZF390 genome sequence against other four *Paenibacillus* genome sequences. (**A**) Synteny analysis of the strains *P. peoriae* ZF390, *P. peoriae* HS311, *P. polymyxa* HY96-2, *P. polymyxa* SQR-21, and *P. kribbensis* PS04 genomes, pairwise alignments of genomes were generated using Mauve. Strain ZF390 genome as the reference genome. Boxes with same color indicate syntenic regions. Boxes below the horizontal strain line indicate inverted regions. Rearrangements are shown with colored lines. The scale is in nucleotides. (**B**) Venn diagram showing the number of shared and unique clusters of orthologous genes.

To estimate the evolutionary distance among these five *Paenibacillus* strains, their whole genome sequences were compared using Mauve (Figure 6A). The alignments between ZF390 and HS311 showed that several gene inversions and a large region of deletion were detectable in *P. peoriae* ZF390. Compared to SQR-21 and HY96-2, a number of gene insertions or deletions and local collinear block (LCB) inversions were also detected in strain ZF390. These results show that large LCB inversions occurred among strain PS04 and the other four strains, and the ZF390 genome is much more similar to that of HS311, supporting the relationship described above (Figure 6A).

Whole genome sequences of these five *Paenibacillus* strains were also compared to identify specific genes in *P. peoriae* ZF390 (Figure 6B). As shown in Figure 6B, there were 3372 core genes present in ZF390 that were shared with strains HS311, SQR-21, HY96-2, and PS04. It was speculated that these orthologous protein-coding genes were relatively conserved in *Paenibacillus*. Additionally, 4122 orthologous genes were shared between ZF390 and HS311, while ZF390 shared 3912 and 3889 orthologous genes with SQR-21 and HY96-2, respectively. Moreover, strain ZF390 shared merely 3652 orthologous genes with PS04, whereas it indicated a higher level of similarity with strain HS311. Furthermore, only two and three unique genes were identified in the genomes of strains ZF390 and PS04, which revealed close relationships among the five selected strains.

### 3.5. Gene Clusters Involved in the Synthesis of Secondary Metabolites

**Figure 7:**
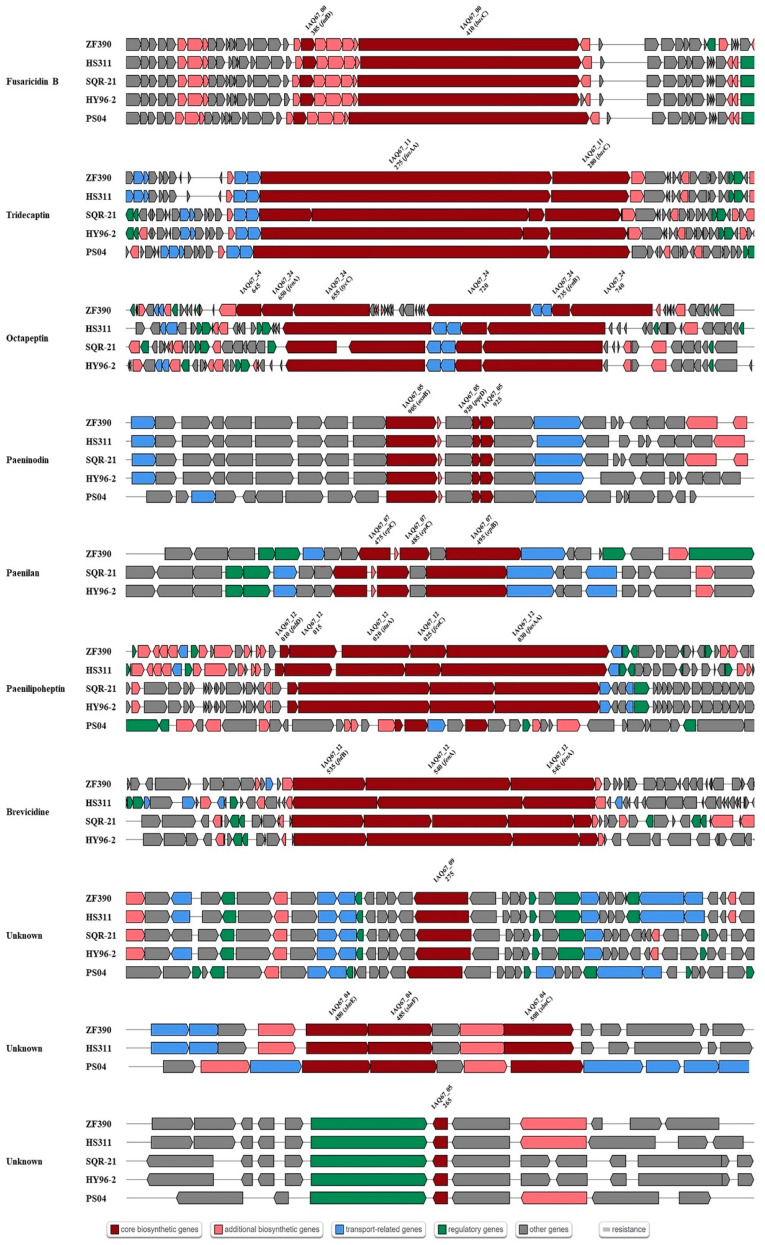
Comparison of gene clusters and core genes involved in antibiotic biosynthesis in strains ZF390 as well as strains HS311, SQR-21, HY96-2, and PS04. Dark red indicates the core biosynthetic genes in different gene clusters among five *Paenibacillus* strains. The core biosynthetic genes are labeled in each gene cluster.

With the aim of identifying the antibiotics that may be produced by ZF390, with its broad antagonistic spectrum, the gene clusters for antibiotic biosynthesis were detected in the ZF390 genomic sequence using the antiSMASH database. Ten gene clusters with different biosynthesis pathways involved in secondary metabolite production were retrieved, including three clusters encoding NRPSs (nonribosomal peptide synthetases), one T1PKS (type Ι polyketide synthases) or NRPS, one TransAT-PKS (trans-acyl transferase polyketide synthetases) or NRPS, one lassopeptide, one siderophere, one lanthipeptide-class-i, one RiPP-like (ribosomally synthesized and post-translationally modified peptides) protein, and an NRPS-like protein (Table 2). Gram-negative bacteria can be suppressed by brevicidine, tridecaptin, paeninodin, and octapeptin while paenilan, fusaricidin B, paeninodin, and octapeptin control Gram-positive bacteria [49,50,51,52,53,54,55]. Meanwhile, fusaricidin B, octapeptin, and paeninodin show an outstanding inhibitory effect against fungal pathogens [49,54,55]. The bioactive spectrum of paenilipoheptin and three unknown antibiotics found in the genome is still unclear (Table 2). The comparison analysis of the above five strains revealed a deficiency of the paenilan synthesis gene cluster in strain HS311. An unknown antibiotic synthesized via the siderophore pathway was not retrieved in SQR-21 and HY96-2. The genome of PS04 did not contain the relevant clusters of brevicidine, octapeptin, and paenilan. Interestingly, however, the gene cluster of paenilipoheptin identified in strain PS04 showed poor similarity with the clusters retrieved in strains ZF390, HS311, SQR-21, and HY96-2 (Figure 7). Furthermore, the gene cluster for the biosynthesis of octapeptin detected in the ZF390 genome had a larger core gene cluster (Figure 7).

### 3.6. Mining for Functional Genes Potentially Associated with Plant–Bacteria Interactions

The key genes related to plant-growth promotion, biofilm formation, and induced systemic resistance were retrieved from *P. peoriae* ZF390 based on the KEGG database, and the identities of these genes among HS311, SQR-21, HY96-2, and PS04 were compared according to the NCBI database. The identities of all homologous sequences between strain ZF390 and the other four strains were higher than 77%. The sequence identities of strain ZF390 exhibited very high similarities with those in strain HS311; concurrently, lower similarities were observed when comparing ZF390 and PS04 (Supplementary Tables S6–S8).

Five key genes related to phosphate solubilization (*phnE*, *phnC*, *pstB*, *pstA*, and *pstC*) and 18 other genes responsible for organic acid biosynthesis were all retrieved from the five *Paenibacillus* strains with high similarity (87.07–99.21%) (Supplementary Table S6). The results demonstrated a latent capacity of ZF390 to promote plant growth. As data shown in Supplementary Table S7 indicate, genes involved in biofilm formation, such as flagellar assembly, non-ribosomal peptide structures, bacterial chemotaxis, and quorum sensing were mined in the genome sequences of five *Paenibacillus* strains. Flagellar structure coding genes, including *fli* gene cluster (*fliACEFGHIJKLMNOPQRY*), *flg* gene cluster (*flgBCDEFGKLM*), gene *flhA* and gene *flhB*, and flagellar motivation coding genes *motA* and *motB*, were located in the genome of strain ZF390. However, gene *fliD* and gene *fliS* were not found in ZF390 genomic sequence. Genes (*fenA*, *fenB*, and *fenC*) related to Fengycin production were also retrieved. The *che* gene cluster (*cheABCDRWXY*) and multiple copies of gene *mcp* were found in five genomes, and the sequence identity exceeded 84.03%. The key gene in AI-2 QS system, *luxS*, and the four genes (*spo0A*, *spo0B*, *spo0F*, and *degU*) involved in biofilm formation pathway were found in genome of strain ZF390, and the sequence identity between ZF390 and HS311 (97.64%) exhibited higher values than those between strain ZF390 with SQR-21 (92.42%), HY96-2 (92.42%), or PS04 (91.06%). Key genes related to the plant-resistance inducers in ZF390 were retrieved in light of previous studies, and the identities of these genes among HS311, SQR-21, HY96-2, and PS04 were compared [7,56]. The results demonstrate that the key genes associated with volatile organic compounds belong to systemic resistance inducers, such as 2,3-butanediol, methanethiol, and isoprene synthesis, were all detected in the genomes of the five strains, with sequence identities exceeding 85% (Supplementary Table S8).

## 4. Discussion

*Pbr* has caused a devastating soft rot disease of cucumbers in China, and chemical agents did not effectively control it; so, biocontrol has been given higher expectations and attention [4]. Chemical methods are commonly used to control soft rot but are not desirable, due to increased resistance and environmental contamination [57,58]. The biocontrol of soft rot through biocontrol strains is one of the safe, effective, and sustainable methods and research hotspots used to control soft rot [59,60,61]. Abundant biocontrol agents were screened through in vitro inhibition tests, for instance, estimating the inhibition of a target pathogen on agar tablets; however, the bottom line for biocontrol is whether it works under production conditions, and numerous researchers have reported no correlation between in vitro inhibition tests and field performance [62].

In this study, *P. peoriae* ZF390 showed a conspicuous alleviation effect on cucumber plants infected with soft rot disease in a greenhouse, representing the good potential of biopesticides or microbial inoculants, even though its effect on the plate assays was not particularly noteworthy (Figure 1 and Supplementary Figure S1). In addition, the antimicrobial properties of strain ZF390 were investigated, demonstrating antagonistic activity against a variety of bacteria and fungi (Figure 2), and the results found were similar to those of previous studies [63,64]. Extracellular enzymes, such as protease (Prt) and cellulase (Cel), produced by strain ZF390 might result in antagonistic action against a wide range of microorganisms (Supplementary Figure S2A,B). Due to its superior antimicrobial activity, the genome of *P. peoriae* ZF390 was completely sequenced to provide insight into its biocontrol mechanisms. Metal-tolerant bacteria could survive in the tissue of plants grown on the metal-polluted soil and perform good adaptability to the environment [65]. Strain ZF390 might be used in a wide range of conditions due to its tolerance to Cd^2+^, Co^2+^, Mn^2+^, Pb^2+^, and Zn^2+^ (Supplementary Figure S2D).

*P. peoriae* was originally recognized as *Bacillus peoriae* and reclassified as a member of the genus *Paenibacillus* after performing an amplified ribosomal DNA restriction analysis, a fatty acid methyl ester analysis, sodium dodecyl sulfate–polyacrylamide gel electrophoresis of whole-cell proteins, API, and other routine phenotypic tests [28,66]. Due to its extreme uniformity, 16S rDNA is rarely recommended for examining the relationships between genera or even deeper taxonomic levels, while multilocus sequence typing (MLST) with a sample of between four- and eight-core gene loci is sufficient for the subdivision of most bacterial species [67]. However, there is a general consensus among taxonomists that all taxonomic information about a bacterium is incorporated into the complete nucleotide sequence of its genome [68]. The average nucleotide identity (ANI) and DNA–DNA hybridization (DDH) are considered significant criteria for species identification. The commonly accepted ANI criteria to set bounds on species is generally 95% [69,70], and the critical value of DDH is typically 70% [71]. Nevertheless, an ANI threshold between 93% and 96% can be considered a fuzzy zone where the intra-genus and interspecies boundary of may fall; the definition of the optimal ANI range for prokaryotic species is still controversial [72]. Previous studies corroborated that the ≥95% ANI cutoff alone is unable to resolve closely related species, and the stringent ≥96% ANI cutoff is more accurate with a better correlation with the 70% DDH cutoff [47,73]. It is highlighted that species assignment based on a single algorithm may not be robust enough.

In silico DNA–DNA hybridization (*is*DDH), determined using the Genome-to-Genome Distance Calculator (Formula 2), is particularly attractive, as it has been shown to be better correlated to the traditional DDH, which is a gold standard for species assignment, than to ANI [43]. Therefore, it is proposed that any genome-based species assignments should be determined by both ANI and *is*DDH, and the pairwise ANI with a ≥96% cutoff and *is*DDH with a ≥70% cutoff have been widely used for precise species identification [47,48]. Based on phenotypic, phylogenetic, and genotypic data, *P. peoriae* ZF390 was subjected to a taxonomic study by using a polyphasic approach. The phylogenetic tree based on eight housekeeping genes revealed an intimate but independent relationship among *P. peoriae*, *P. polymyxa*, and *P. kribbensis* (Figure 5). The ANI and *is*DDH values for strains ZF390 and HS311 were 96.61% and 71.80%, respectively, which were considerably higher than the values for ZF390 with the selected *P. polymyxa* and *P. kribbensis* strains (Supplementary Figure S5).

In contrast to the other four strains, the genome of ZF390 was larger, comprising more plasmids, but with a slightly smaller GC content, and abundant CDSs were predicted (Supplementary Table S3). The analysis of the whole ZF390 genome revealed an impressive capability to produce a diverse spectrum of different secondary metabolites aimed at suppressing harmful phytopathogens. In total, 10 gene clusters (Table 2) representing more than 8.7% of the genome were devoted to synthesizing antimicrobial metabolites, approximately the same proportion as the distinguished biocontrol agent *B. amyloliquefaciens* FZB42 [74].

On the basis of antiSMASH database, comparisons of the genes and gene clusters related to antibiotic synthesis demonstrated differences among the five *Paenibacillus* strains (Table 2 and Figure 7). Regarding the control of bacteria, five antibacterial metabolites (brevicidine, tridecaptin, paenilan, fusaricidin B, paeninodin, and octapeptin) were predicted to be produced by *P. peoriae* ZF390. Brevicidine, a bacterial non-ribosomally-produced cyclic lipododecapeptide, which has low hemolytic activity and cytotoxicity, displays potent antimicrobial activity against Gram-negative pathogens [75]. Brevicidine B was discovered from the producer strain of brevicidine, *Brevibacillus laterosporus* DSM 25. Recently, researchers found that brevicidine B, compared with an earlier reported member of the brevicidine family, has a broadened antimicrobial spectrum caused by its increased membrane-disruptive capacity against Gram-positive pathogens [52]. The strong antibacterial activity of strain ZF390 might be associated with the production of brevicidine; however, its capability of producing brevicidine B should be further investigated. Tridecaptin is another type of non-ribosomal antibacterial peptide (NRAP) with effective activity against highly problematic strains of Gram-negative bacteria and low levels of resistance development with the potential for safe administration [76]. All five Paenibacillus strains were found to contain tridecaptin-related gene clusters, but more core biosynthetic genes were predicted in SQR-21 and HY96-2 than in the other three strains (Figure 7). With purification and structure analysis, paenilan was identified as a novel class-Ⅰ antibiotic, a ribosomally synthesized peptide that exhibits antimicrobial activity against Gram-positive bacteria and is well conserved in different *Paenibacillus* sp. isolated from globally distant places [53]. The core genes *epiB* and *epiC* and biosynthesis cluster were observed in the genomes of ZF390, SQR-21, and HY96-2, while no genes or gene clusters involved in the synthesis of paenilan were found on the chromosomes of HS311 and PS04 (Table 2). Fusaricidin B, paeninodin, and octapeptin not only possess the antibacterial activity, but also impede the growth of phytopathogenic fungi. According to former reports on its particularly antagonistic activity against Gram-positive bacteria and plant pathogenic fungi, fusaricidin, generated by a non-ribosomal peptide synthetase (NRPS), seems to have immense potential for industrial use [77,78]. Fusaricidin B that was possibly produced by strain ZF390 reported excellent germicidal activity against *Candida albicans* and *Saccharomyces cerevisiae* [54]. Octapeptins, which are structurally similar to polymyxins, are lipopeptide antibiotics possessing much broader antimicrobial activity against Gram-negative and Gram-positive bacteria, yeast, fungi, and even protozoa, whereas polymyxins are only active against Gram-negative bacteria [55]. The core gene region belonging to the synthesis gene cluster of octapeptin predicted in strain ZF390 was much larger than that in HS311, SQR-21, and HY96-2; the octapeptin cluster was not found in strain PS04 (Figure 7). Paeninodin, a new lasso peptide, belongs to the ribosomally synthesized and post-translationally modified peptides (RiPPs) that exhibit a wide range of antimicrobial or antiviral activities, and the synthesis cluster was found in all five strains [79]. Paenilipoheptin was recently identified via the genome mining of *P. polymyxa* E681, and its antimicrobial activity spectrum remains to be elucidated [23,80].

Bacteria belonging to the genus *Paenibacillus* have been isolated from a variety of environments, and the majority of them are found in soil, demonstrating benefits for plants with mutual interactions. They are considered predominant plant-growth-promoting rhizobacteria (PGPR) and are widely used for agricultural applications [81,82]. Phosphorus is a significant macronutrient for plant nutrition, but its unique characteristics of high fixation and poor solubilization in soil lead to the overuse of chemical phosphorus fertilizers applied to agricultural land, causing severe environmental damage [83,84]. By inoculating fields with phosphorus-solubilizing microorganisms, such as *Paenibacillus*, the application of chemical phosphorus fertilizer could be reduced, and the principal mechanism of microorganisms to make phosphorus available to plants could be performed by producing organic acids [85]. A differential gene expression study revealed an extremely intricate transcriptional response of *P. sonchi* SBR5 to soluble and insoluble phosphorus sources, suggesting that the production of organic acids might be the most vital strategy utilized by SBR5 to perform phosphorus solubilization [86]. An evident and clear dissolution halo can be observed around the *P. polymyxa* inoculum grown on a solid NBRIP medium, in which Ca_3_(PO_4_)_2_ is the sole phosphate source [87]. According to the comparative genomic analysis, 23 genes related to phosphorus solubilization were retrieved in the ZF390 genome, some of which encode organic acid production (Supplementary Table S5). However, the main genes (*gcd* and *gad*) responsible for the production of gluconic acid and its conversion were not detected in the genome of ZF390. Similarly, ZF390 does not carry the complete phosphonate gene cluster (*phn*), except for *phnC* and *phnE*, which were deemed a gene gain and loss event during evolution [88]. In addition, the ZF390 genome possesses the *pst* operon (*pstC*, *pstA*, and *pstB*) that integrates the phosphate-specific transport system. Taken together, *P. peoriae* ZF390 is a potential PGPR that may become an additive in biofertilizer.

Endospore-forming bacteria such as *Paenibacillus* have the capacity to survive for long periods in the soil under adverse environmental conditions, which is appropriate for field inoculation [85]. PGPR plays a crucial role in plant growth promotion by effectively colonizing plant roots. Bacteria in natural environments persist by forming biofilms, which are highly structured, surface-attached communities of cells encased in a self-produced extracellular matrix [89]. A series of genes related to flagellar assembly, non-ribosomal peptide structures, bacterial chemotaxis, and quorum sensing play increasingly important roles in biofilm formation. *Paenibacillus* performs two forms of flagellar-driven motility: swarming on a surface and swimming in liquid culture; swarming motility is crucial during root colonization or for rapid colonization of a specific tissue [20,90]. A threshold amount of power is needed for an organism to swarm, and hyper-flagellation, a dramatic augmentation in flagellum density, may contribute to a cumulative increase in power [90]. According to the KEGG database and comparative genomics results, a complete flagellar assembly gene cluster was revealed in the ZF390 genome, including, but not limited to, the *fliC* gene encoding the filament, the *flgE*, *flgK*, and *flgL* genes encoding the hook, and multitudinous genes encoding the basal structure [91,92]. Recent research reported that *P. polymyxa* generates energy through the mechanism of degrading phospholipids to create sn-glycerol 3-phosphate and feed it into the glycolysis/pentose phosphate pathway, which appears likely to be necessary for swarming motility, including flagellar rotation [90]. However, the applicability of this mechanism in ZF390 needs to be further explored. In addition to multicellular rafts of highly flagellated cells, the lipopeptide surfactants or non-ribosomal peptides secreted by bacteria act as wetting agents, providing a mechanism to reduce surface tension between the substrate and the bacterial cells, helping them spread rapidly over surfaces [90,93,94,95]. Except for the non-ribosomal peptide biosynthesis gene clusters identified in the antiSMASH database, several genes belonging to fengycin synthesis were also detected in the genome of ZF390. The presence of these substances distinctly provides the essential requirement for biofilm swarms on plant roots.

Bacterial chemotaxis, a biased movement in chemical gradients, is a motility-based response that generally enhances the efficiency of environmental colonization by motile bacteria [96]. Chemoreceptors called methyl-accepting chemotaxis proteins (MCPs) detect various environmental signals to stimulate the chemotactic response [97]. The basic molecular mechanism of chemotaxis is generally conserved in bacteria, and it was systematically studied in *Escherichia coli*, revealing only a single chemotaxis operon in its genome [96,98,99]. Nevertheless, multiple parallel chemotaxis systems and a large number of chemoreceptors have been found in motile bacterial species, which is likely to provide a competitive advantage for surviving in the soil, as well as competition for root-surface colonization [100]. We found multiple copies of genes, especially the *mcp* gene, involved in chemotaxis in the genome of ZF390. Thus, we propose that chemotaxis may play a crucial role in the colonization of ZF390.

Moreover, bacterial biofilm formation is often regulated by quorum sensing (QS), which is a cell-communication mechanism that depends on population density [101]. To date, two typical QS systems using an autoinducing peptide (AIP) or an autoinducer-2 (AI-2) as a signal molecule have been reported in Gram-positive bacteria [102]. The *luxS* gene, a key regulatory gene of the AI-2-mediated QS system, has been confirmed to impact the biomass and morphology of biofilms formed by *Paenibacillus* species. In *P. polymyxa* HY96-2, *luxS* improved biocontrol efficacy by promoting its biofilm formation ability [103]. On the basis of recent studies, we speculate that *luxS* can positively regulate biofilm formation, but this assumption needs more experimental arguments [103,104]. The biofilm formation pathway genes *spo0A*, *spo0B*, *apo0F*, and *degU* were found in the ZF390 genome when searching for QS genes, suggesting that two pathways may be involved in ZF390 [56]. From the above results, it is possible to deduce that ZF390 could colonize well in the rhizosphere by forming biofilms, but the colonization abilities of the five strains might differ because the variety, amount, and expression levels of these genes related to biofilm formation are different.

Many beneficial rhizobacteria including members of *Paenibacillus* can trigger ISR when present in sufficiently high population densities [85]. Systemic resistance inducers, such as volatile organic compounds (VOCs), including methanethiol, isoprene, 2,3-butanediol and butyl acetate, are generated and released into the surrounding environment by biocontrol agents, offering protection against phytopathogens and insect herbivores [85]. *P. polymyxa* E681 was interpreted to use VOC elicitors to protect *Arabidopsis thaliana* plants against the bacterium *P. syringae* pv. *maculicola* via the primed transcription of salicylic acid, jasmonic acid, and ethylene-signaling genes [105]. In previous studies, key genes associated with VOC production were investigated in *P. polymyxa* strains HY96-2, ZF129, and ZF197 [56,88]. The genes responsible for inducers (2,3-butanediol, methanethiol and isoprene) in strains ZF390 and HS311 exhibited 93% identity, which is higher than the identity observed among strains SQR-21, HY96-2, and PS04. These results deduced a similar capability of systemic resistance in these five strains with varying effectiveness owing to the variations in the related genes.

## 5. Conclusions

*P. peoriae* ZF390, which was obtained from cucumber rhizosphere soil, exhibited an extraordinary effect on controlling cucumber soft rot caused by *Pbr* and exhibited broad-spectrum antagonistic activities against 16 distinct bacterial and fungal phytopathogens. Based on the polyphasic analyses combined with phenotypic, phylogenetic, and genotypic data, strain ZF390 was identified as *P. peoriae*, which is closely related to *P. polymyxa* and *P. kribbensis*. According to the antiSMASH database, strain ZF390 possesses miscellaneous secondary metabolite biosynthesis clusters with biocontrol functions, including brevicidine, fusaricidin B, octapeptin, paenilan, paenilipoheptin, paeninodin, tridecaptin, and three other unknown antibiotics, demonstrating preeminent antimicrobial activity. Based on the comparative genomic analysis and phosphate solubilization assay, strain ZF390 may perform as a potential PGPR. In addition, many genes associated with colonization, such as flagellar assembly, bacterial chemotaxis, quorum sensing, and non-ribosomal peptide structures, were detected in the ZF390 genome and shared high homology with *P. peoriae* strain HS311. Overall, these properties indicate that ZF390 is a promising biocontrol agent for further application in agricultural fields.

## Figures and Tables

**Table 1:** Control efficiency of antagonistic strains against cucumber bacterial soft rot disease caused by *Pbr*. Data with different lowercase letters indicated significant difference at *p* < 0.05 level.

Treatment	Disease Index	Control Effect (%)
ZF115	76.00 ± 6.93 bcd	19.72 ± 7.32 defg
ZF119	77.33 ± 4.62 abc	18.31 ± 4.88 efg
ZF185	88.00 ± 6.93 ab	7.05 ± 7.32 g
ZF194	34.67 ± 4.62 fg	63.38 ± 4.88 ab
ZF278	81.33 ± 12.86 ab	14.09 ± 13.58 g
ZF390	21.33 ± 11.55 g	77.47 ± 12.20 a
ZF402	58.67 ± 14.05 e	38.03 ± 14.84 cd
ZF405	78.67 ± 6.11 abc	16.90 ± 6.45 fg
ZF428	40.00 ± 8.00 f	57.75 ± 8.45 b
ZF429	85.33 ± 6.11 ab	9.86 ± 6.45 g
ZF436	60.00 ± 12.00 de	36.62 ± 12.68 cde
ZF448	78.67 ± 4.62 abc	16.90 ± 4.88 fg
ZF450	76.00 ± 12.00 bcd	19.72 ± 12.68 defg
ZF453	62.67 ± 16.17 cde	33.81 ± 17.08 cdef
Zhongshengmycin	49.33 ± 2.31 ef	47.89 ± 2.44 bc
Control	94.67 ± 6.11 a	-

**Table 2:** Comparative analysis of secondary metabolites clusters in strains ZF390, HS311, SQR-21, HY96-2, and PS04.

Antibiotic Name	Type	Core Gene Clusters	Size	Position	Bioactive Spectrum	ZF390	HS311	SQR-21	HY96-2	PS04
Brevicidine	T1PKS, NRPS	*fen*A, *ftd*B	761,14 bp	2792116–2868229	G^-^bacteria	IAQ67_12465- IAQ67_12650	ABE82_12870-ABE82_13050	PPSQR21_024840-PPSQR21_025180	C1A50_2551-C1A50_2581	NA
Fusaricidin B	NRPS	*fad*D, *bac*C	68,481 bp	62571– 131051	Fungi, G^+^ bacteria	IAQ67_00290- IAQ67_00510	ABE82_00305-ABE82_00500	PPSQR21_000590-PPSQR21_001000	C1A50_0064-C1A50_0108	FOA15_RS06155- FOA15_RS06360
Octapeptin	NRPS	*fen*AB, *tyc*C	115,828 bp	5324980–5440807	Fungi, bacteria	IAQ67_24570- IAQ67_24795	ABE82_22040-ABE82_22245	PPSQR21_044420-PPSQR21_044780	C1A50_4551-C1A50_4603	NA
Paenilan	Lanthipeptide- class-i	*epi*BC	27,007 bp	1620879–1647885	G^+^ bacteria	IAQ67_07435- IAQ67_07530	NA	PPSQR21_015740-PPSQR21_015950	C1A50_1599-C1A50_1620	NA
Paenilipoheptin	TransAT-PKS, NRPS	*fab*D, *itu*A, *fen*C, *fus*AA	82,343 bp	2634231–2716573	NA	IAQ67_11935- IAQ67_12110	ABE82_12325-ABE82_12500	PPSQR21_009520-PPSQR21_009940	C1A50_0993-C1A50_1036	FOA15_RS17665- FOA15_RS17835
Paeninodin	Lassopeptide	*asn*B, *pqq*D	24,119 bp	1301862–1325980	Fungi, bacteria, virus	IAQ67_05860- IAQ67_05975	ABE82_06065-ABE82_06175	PPSQR21_012140-PPSQR21_012370	C1A50_1276-C1A50_1299	FOA15_RS11780- FOA15_RS11890
Tridecaptin	NRPS	*fus*AA, *bac*C	92,515 bp	2419321–2511835	G^-^bacteria	IAQ67_11205- IAQ67_11360	ABE82_11520-ABE82_11675	PPSQR21_022460-PPSQR21_022870	C1A50_2315-C1A50_2359	FOA15_RS17145- FOA15_RS17325
Unknown	Siderophore	*sbn*CEF	17,402 bp	999368– 1016769	NA	IAQ67_04460- IAQ67_04525	ABE82_04615-ABE82_04685	NA	NA	FOA15_RS16945- FOA15_RS17000
Unknown	RiPP-like	NA	10,237 bp	1167935–1178171	NA	IAQ67_05235- IAQ67_05290	ABE82_05390-ABE82_05445	PPSQR21_010820-PPSQR21_010940	C1A50_1133-C1A50_1145	FOA15_RS11190- FOA15_RS11225
Unknown	NRPS-like	NA	43,119 bp	1991107–2034225	NA	IAQ67_09200- IAQ67_09365	ABE82_09545-ABE82_09710	PPSQR21_018700-PPSQR21_019060	C1A50_1915-C1A50_1953	FOA15_RS15165- FOA15_RS15330

NA = not available.

## Data Availability

The complete genome sequences of *P. peoriae* ZF390 were deposited in NCBI GenBank under the accession numbers CP061172.1, CP061173.1, CP061174.1, and CP061175.1, respectively. The Sequence Read Archive (SRA) submission was completed under the accession number SRR19570818. The strain ZF390 was also deposited in the China General Microbiological Culture Collection Center (CGMCC) for Type Culture Collection under the accession number 20322.

## Supplementary Materials

The following supporting materials can be downloaded at: https://www.mdpi.com/article/10.3390/biology11081172/s1, Figure S1: Inhibition zone of antagonistic strains against *Pbr* in plate assays. (A): ZF115; (B): ZF119; (C): ZF185; (D): ZF194; (E): ZF278; (F): ZF390; (G): ZF402; (H): ZF405; (I): ZF428; (J): ZF429; (K): ZF436; (L): ZF448; (M): ZF450; (N): ZF453. Figure S2: Plate assays for production of cellulase (A), protease (B) and phosphatase (C) by *Paenibacillus peoriae* ZF390 and heavy metal resistance of strain ZF390 (D). Figure S3: Colonization of the strain ZF390 in different types of soils. Figure S4: General characteristics of *Paenibacillus peoriae* ZF390. (A): Image of ZF390 colony morphology; (B): Image of ZF390 cells using transmission electron microscopy. Figure S5: Percentage of average nucleotide indentities (ANI) and in silico DNA-DNA hybridization (*is*DDH) among the selected *Paenibacillus* genomes. Table S1: Physiological and biochemical characteristics of strain ZF390. Table S2: Genome statistics. Table S3: Genomic features of *P. peoriae* ZF390 and other Paenibacillus strains. Table S4: Number of genes associated with general COG functional categories. Table S5: Strains and housekeeping gene locus tag information used for the phylogenetic tree construction in this study. Table S6: Comparison of genes involved in plant growth promotion between strains ZF390, HS311, SQR-21, HY96-2 and PS04. Table S7: Comparison of genes mostly involved in biofilm formation between strains ZF390, HS311, SQR-21, HY96-2, and PS04. Table S8: Comparison of genes involved in synthesis of resistance inducers between strains ZF390, HS311, SQR-21, HY96-2, and PS04.
