# Supplementary material for: Comparative Genomics Insights into a Novel Biocontrol Agent Paenibacillus peoriae Strain ZF390 against Bacterial Soft Rot
Source: Biology (Basel). 2022 Aug 4;11(8):1172. doi: 10.3390/biology11081172 (PMC9404902; doi:10.3390/biology11081172)
Supplement: Supplementary file 1 [file biology-11-01172-s001.zip › Supplementary Figures.pdf]

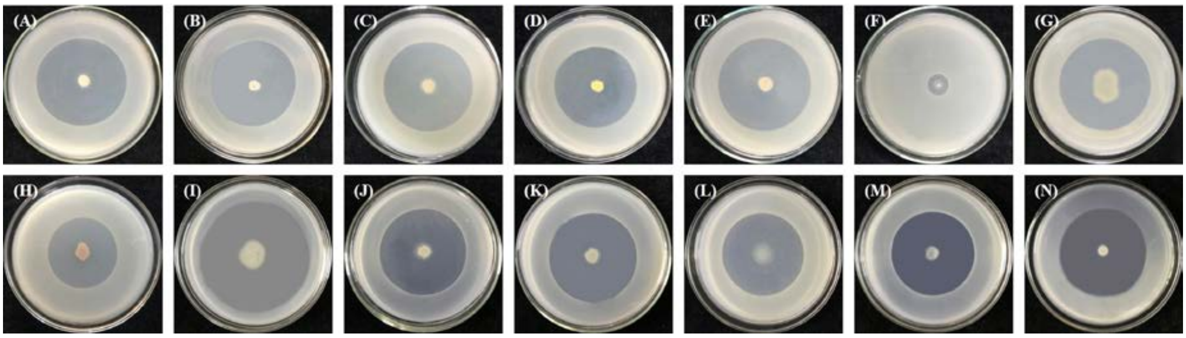

Figure S1. Inhibition zone of antagonistic strains against *Pbr* in plate assays. (A): ZF115; (B): ZF119; (C): ZF185; (D): ZF194; (E): ZF278; (F): ZF390; (G): ZF402; (H): ZF405; (I): ZF428; (J): ZF429; (K): ZF436; (L): ZF448; (M): ZF450; (N): ZF453.

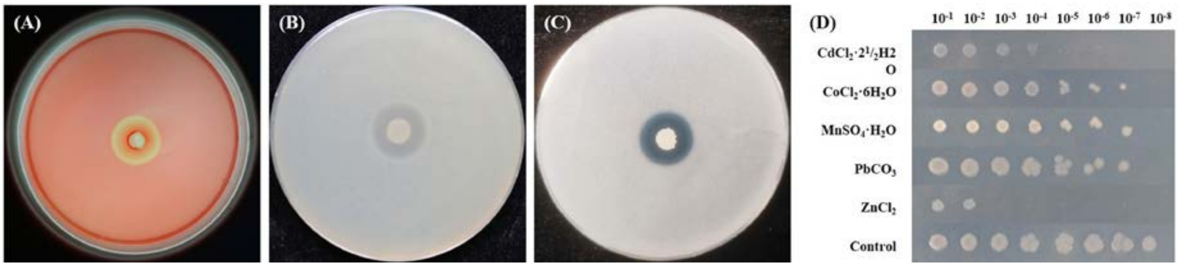

Figure S2. Plate assays for production of cellulase (A), protease (B) and phosphatase (C) by *Paenibacillus peoriae* ZF390 and heavy metal resistance of strain ZF390 (D).

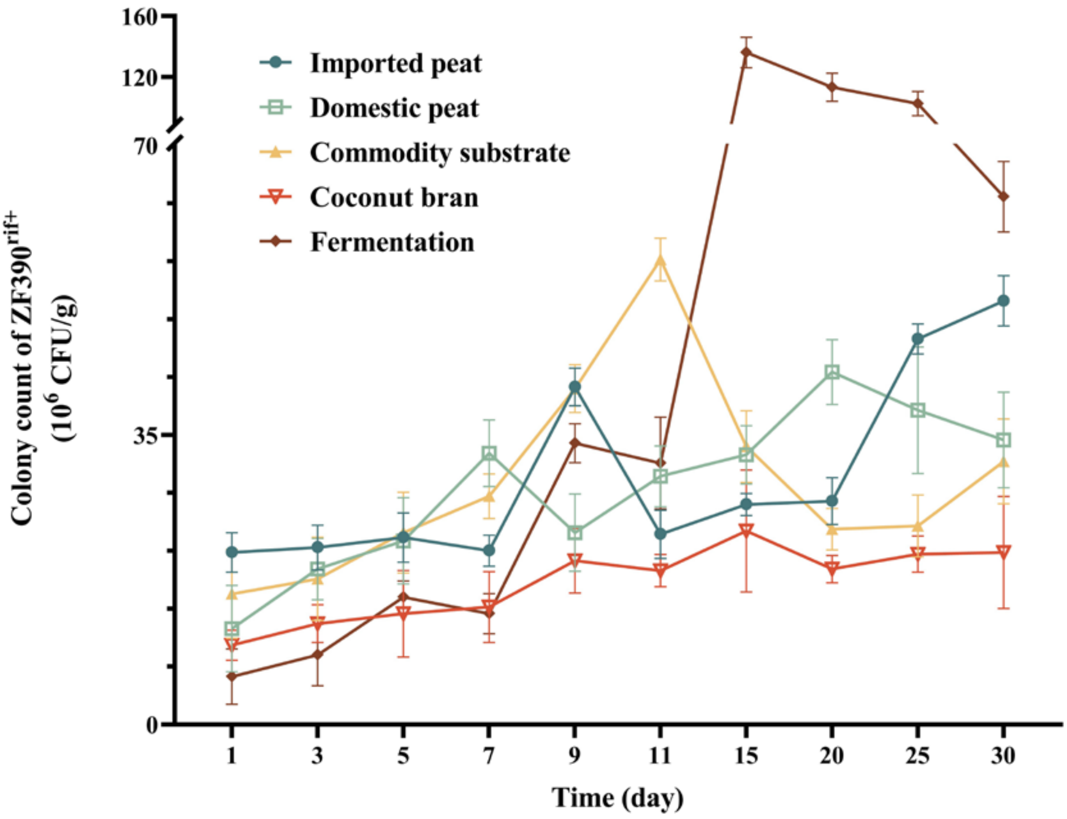

Figure S3. Colonization of the strain ZF390 in different types of soils.

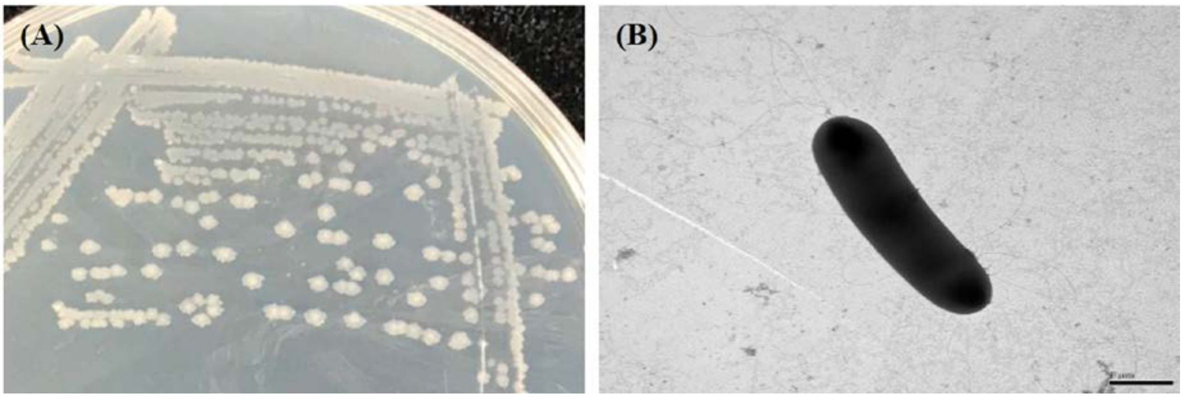

Figure S4. General characteristics of *Paenibacillus peoriae* ZF390. (A): Image of ZF390 colony morphology; (B): Image of ZF390 cells using transmission electron microscopy.

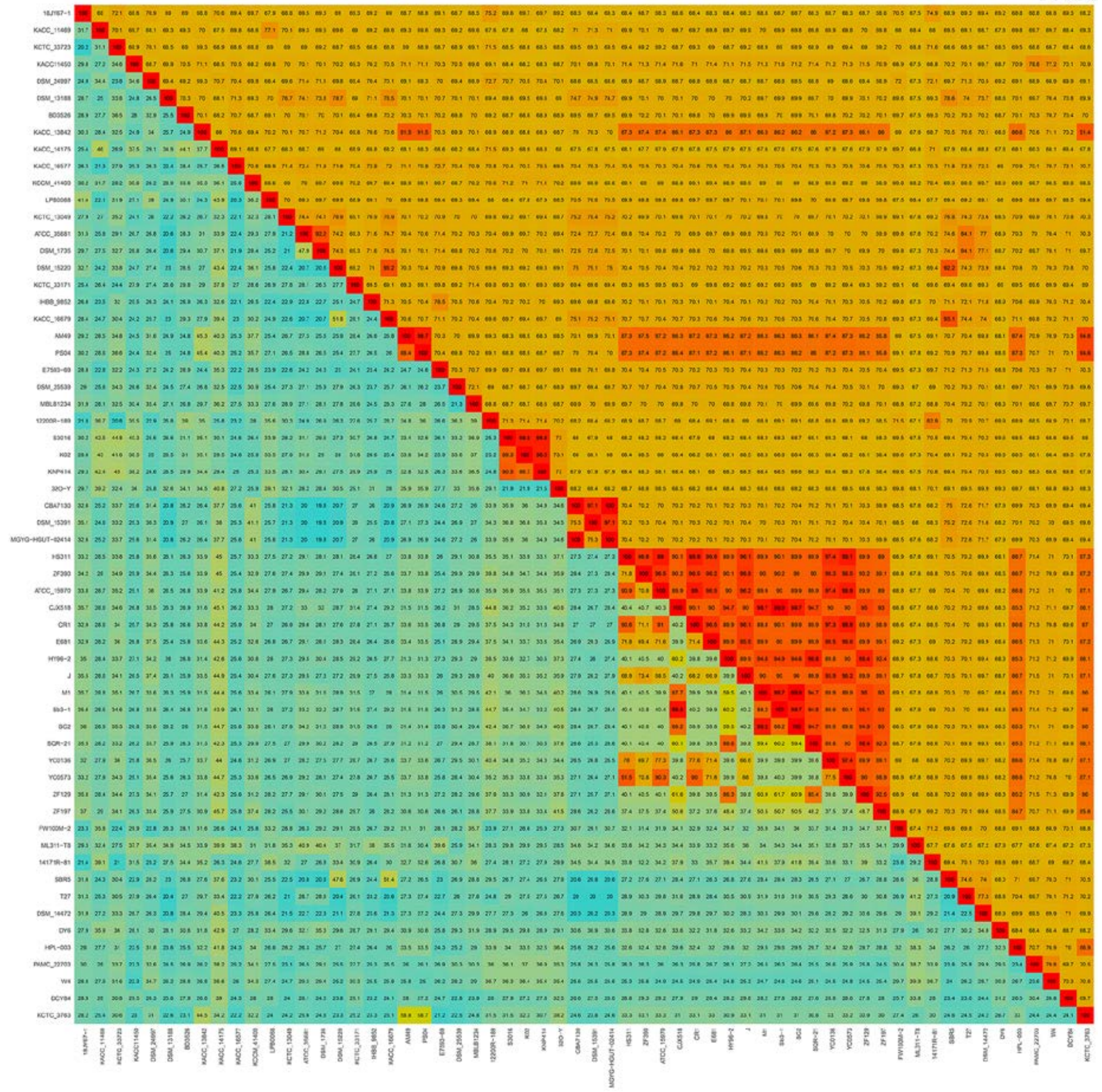

Figure S5. Percentage of average nucleotide indentities (ANI) and in silico DNA-DNA hybridization (*is*DDH) among the selected *Paenibacillus* genomes. .
