# Supplementary material for: Comparative Genomics Insights into a Novel Biocontrol Agent Paenibacillus peoriae Strain ZF390 against Bacterial Soft Rot
Source: Biology (Basel). 2022 Aug 4;11(8):1172. doi: 10.3390/biology11081172 (PMC9404902; doi:10.3390/biology11081172)
Supplement: Supplementary file 1 [file biology-11-01172-s001.zip › Supplementary Table S1.pdf]

Supplementary Table S1 Physiological and biochemical characteristics of strain ZF390.

Characteristic	ZF390	KCTC 3763^T
Casein hydrolysis	+	+
Arginine dihydrolase	-	-
Indole production	-	-
Gelatin liquefaction	-	+
Nitrate reduction	-	-
Acid produced from:		
Glycerol	+	+
D-arabinose	-	-
L-arabinose	+	+
Ribose	+	+
D-xylose	+	+
Adonitol	-	-
Methylxyloside	+	+
D-fructose	+	+
Mannitol	+	+
Methyl-D-mannoside	+	+
Salicin	+	+
Maltose	+	+
D-trehalose	-	-
Gentiobiose	+	+
2-keto-D-gluconate	-	-
5-keto-D-gluconate	-	-
Rhamnose	-	-
D-turanose	+	-
N-acetylglucosamine	+	+
