# Supplementary material for: Comparative Genomics Insights into a Novel Biocontrol Agent Paenibacillus peoriae Strain ZF390 against Bacterial Soft Rot
Source: Biology (Basel). 2022 Aug 4;11(8):1172. doi: 10.3390/biology11081172 (PMC9404902; doi:10.3390/biology11081172)
Supplement: Supplementary file 1 [file biology-11-01172-s001.zip › Supplementary Table S2.pdf]

Supplementary Table S2 Genome statistics.

Attribute	Chromosome value	pPlas1 value	pPlas2 value	pPlas3 value
GenBank sequence	CP061172.1	CP061173.1	CP061174.1	CP061175.1
Genome size (bp)	6193667	137220	16869	36233
GC content (%)	44.96	45.58	45.12	41.66
Predicted genes	5662	169	16	43
Protein coding genes	5353	163	16	42
Ribosomal RNA	40	-	-	-
Transfer RNA	95	6	-	-
Other RNA	4	-	-	-
Pseudo genes	170	-	-	1
GIs number	17	-	-	-
Prophage number	5	-	-	-
CRISPR number	11	-	-	-
Genes assigned to KEGGs	5402			
Genes assigned to GOs	3754			
Genes assigned to NR	5645			
Genes assigned to TCDB	481			
Genes assigned to Pfam	3754			
Genes assigned to Swiss-Prot	2296			
Genes assigned to CAZy	342			
