# Supplementary material for: Comparative Genomics Insights into a Novel Biocontrol Agent Paenibacillus peoriae Strain ZF390 against Bacterial Soft Rot
Source: Biology (Basel). 2022 Aug 4;11(8):1172. doi: 10.3390/biology11081172 (PMC9404902; doi:10.3390/biology11081172)
Supplement: Supplementary file 1 [file biology-11-01172-s001.zip › Supplementary Table S3.pdf]

Supplementary Table S3 Genomic features of *P. peoriae* ZF390 and other *Paenibacillus* strains.

Features	P. peoriae ZF390	P. peoriae HS311	P. polymyxa SQR-21	P. polymyxa HY96-2	P. kribbensis PS04
GeneBank sequence	CP061172.1	CP011512.1	CP006872.1	CP025957.1	CP041731.1
Size (Mb)	6.38	6.22	5.83	5.75	5.74
GC content (%)	44.99	45.47	45.60	45.60	46.80
Replicons	One chromosome<br>Three plasmids	One chromosome<br>One plasmid	One chromosome	One chromosome	One chromosome
Total genes	5,890	5,537	5,071	4,943	5,094
Predicted no. of CDS	5,574	5,265	4,801	4,648	4,798
Ribosomal RNA	40	39	39	42	30
Transfer RNA	101	100	111	110	92
Other RNA	4	4	4	4	4
Pseudo genes	171	129	116	139	170
