# Supplementary material for: Comparative Genomics Insights into a Novel Biocontrol Agent Paenibacillus peoriae Strain ZF390 against Bacterial Soft Rot
Source: Biology (Basel). 2022 Aug 4;11(8):1172. doi: 10.3390/biology11081172 (PMC9404902; doi:10.3390/biology11081172)
Supplement: Supplementary file 1 [file biology-11-01172-s001.zip › Supplementary Table S4.pdf]

Supplementary Table S4 Number of genes associated with general COG functional categories.

Description		Chromosome value	pPlas1 value	pPlas2 value	pPlas3 value	Total value
**Cellular processes and signaling**		**1028**	**7**	**3**	**3**	**1041**
D	Cell cycle control, Cell division, chromosome partitioning	66	0	0	0	66
M	Cell wall/membrane biogenesis	225	3	1	0	229
N	Cell motility	85	0	1	0	86
O	Posttranslational modification, protein turnover, chaperones	158	1	0	0	159
T	Signal transduction mechanisms	304	0	0	0	304
U	Intracellular trafficking and secretion	44	0	0	2	46
V	Defense mechanisms	133	3	1	1	138
W	Extracellular structures	9	0	0	0	9
Z	Cytoskeleton	4	0	0	0	4
**Information storage and processing**		**932**	**12**	**2**	**4**	**950**
A	RNA processing and modification	2	0	0	0	2
J	Translation, ribosomal structure and biogenesis	293	2	0	0	295
K	Transcription	478	2	1	1	482
L	Replication, recombination and repair	159	8	1	3	171
**Metabolism**		**1855**	**12**	**0**	**2**	**1869**
C	Energy production and conversion	166	0	0	0	166
E	Amino acid transport and metabolism	353	1	0	1	355
F	Nucleotide transport and metabolism	106	6	0	0	112
G	Carbohydrate transport and metabolism	503	1	0	1	505
H	Coenzyme transport and metabolism	222	4	0	0	226
I	Lipid transport and metabolism	122	0	0	0	122
P	Inorganic ion transport and metabolism	249	0	0	0	249

Q	Secondary metabolites biosynthesis, transport and catabolism	87	0	0	0	87
X	Mobilome; prophages, transposons	47	0	0	0	47
Poorly characterized		**688**	**4**	**1**	**2**	**695**
R	General function prediction only	446	3	1	1	451
S	Function unknown	242	1	0	1	244
Not in COGs		432	7	0	0	439
