# Supplementary material for: Comparative Genomics Insights into a Novel Biocontrol Agent Paenibacillus peoriae Strain ZF390 against Bacterial Soft Rot
Source: Biology (Basel). 2022 Aug 4;11(8):1172. doi: 10.3390/biology11081172 (PMC9404902; doi:10.3390/biology11081172)
Supplement: Supplementary file 1 [file biology-11-01172-s001.zip › Supplementary Table S5.pdf]

Supplementary Table S5 Strains and housekeeping genes locus tag information used for the phylogenetic tree construction in this study.

Strain	*gyrB*	*rpoD*	*rplA*	*atpD*	*holB*	*rho*	*dnaA*	*rpsA*
Paenibacillus alvei DSM 29^T	PAV_RS09320	PAV_RS15955	PAV_RS06775	PAV_RS07565	PAV_RS09385	PAV_RS10040	PAV_RS09295	PAV_RS13070
Paenibacillus beijingensis DSM 24997^T	VN24_10220	VN24_19405	VN24_12580	VN24_08235	VN24_10290	VN24_10930	VN24_10195	VN24_24850
Paenibacillus chitinolyticus NBRC 15660^T	PCH01S_RS28520	PCH01S_RS13365	PCH01S_RS27990	PCH01S_RS23965	PCH01S_RS27620	PCH01S_RS27275	PCH01S_RS28495	PCH01S_RS08855
Paenibacillus ehimensis NBRC 15659^T	PEH01S_RS27300	PEH01S_RS08230	PEH01S_RS20985	PEH01S_RS25465	PEH01S_RS28245	PEH01S_RS25540	PEH01S_RS27325	PEH01S_RS12040
Paenibacillus elgii NBRC 100335^T	PEL01S_RS23625	PEL01S_RS19625	PEL01S_RS25250	PEL01S_RS30385	PEL01S_RS30940	PEL01S_RS29105	PEL01S_RS23600	PEL01S_RS09150
Paenibacillus glucanolyticus DSM 5162^T	A3958_09445	A3958_17425	A3958_06690	A3958_07450	A3958_09520	A3958_10050	A3958_09420	A3958_19510
Paenibacillus graminis DSM 15220^T	PGRAT_00030	PGRAT_24795	PGRAT_29410	PGRAT_30150	PGRAT_00100	PGRAT_00610	PGRAT_00005	PGRAT_20965
Paenibacillus helianthi P26E^T	A3844_RS26635	A3844_RS04815	A3844_RS20340	A3844_RS15485	A3844_RS20515	A3844_RS23100	A3844_RS26610	A3844_RS12415
Paenibacillus kribbensis AM49^T	B4V02_25205	B4V02_08530	B4V02_02815	B4V02_02015	B4V02_25135	B4V02_24555	B4V02_25230	B4V02_10395
Paenibacillus macerans NBRC 15307^T	PMA02S_RS30570	PMA02S_RS26285	PMA02S_RS16265	PMA02S_RS30935	PMA02S_RS19245	PMA02S_RS24235	PMA02S_RS30545	PMA02S_RS23730
Paenibacillus pabuli NBRC 13638^T	PPA03S_RS26305	PPA03S_RS23730	PPA03S_RS29985	PPA03S_RS08350	PPA03S_RS29675	PPA03S_RS30515	PPA03S_RS26330	PPA03S_RS25735
Paenibacillus peoriae KCTC 3763^T	KQI_RS0110920	KQI_RS0103695	KQI_RS0120930	KQI_RS0125435	KQI_RS0124435	KQI_RS0109780	KQI_RS0110945	KQI_RS0105555
Paenibacillus peoriae ZF390	IAQ67_00030	IAQ67_19485	IAQ67_25150	IAQ67_25910	IAQ67_00100	IAQ67_00730	IAQ67_00005	IAQ67_17705
Paenibacillus polymyxa DSM 36^T	G7035_08355	G7035_17095	G7035_11475	G7035_10705	G7035_08285	G7035_07695	G7035_08380	G7035_18885
Paenibacillus riograndensis SBR5^T	PRIO_RS00030	PRIO_RS25495	PRIO_RS30270	PRIO_RS31100	PRIO_RS00100	PRIO_RS00600	PRIO_RS00005	PRIO_RS22150
Paenibacillus xylanilyticus LMG 21957^T	HP552_RS03245	HP552_RS21925	HP552_RS02505	HP552_RS17300	HP552_RS05875	HP552_RS02930	HP552_RS03220	HP552_RS23540
Paenibacillus yonginensis DCY84^T	AWM70_RS19015	AWM70_RS05900	AWM70_RS16640	AWM70_RS17380	AWM70_RS18945	AWM70_RS18420	AWM70_RS19040	AWM70_RS09900
