# Supplementary material for: Comparative Genomics Insights into a Novel Biocontrol Agent Paenibacillus peoriae Strain ZF390 against Bacterial Soft Rot
Source: Biology (Basel). 2022 Aug 4;11(8):1172. doi: 10.3390/biology11081172 (PMC9404902; doi:10.3390/biology11081172)
Supplement: Supplementary file 1 [file biology-11-01172-s001.zip › Supplementary Table S6.pdf]

Supplementary Table S6 Comparison of genes involved in plant growth promotion between strains ZF390, HS311, SQR-21, HY96-2 and PS04.

Trait	Gene name	ZF390	HS311		SQR-21		HY96-2		PS04	
		Locus tag	Locus tag	Identity (%)	Locus tag	Identity (%)	Locus tag	Identity (%)	Locus tag	Identity (%)
Phosphate solubilization	*phnE*	IAQ67_24275	ABE82_21690	97.31	PPSQR21_043880	92.28	C1A50_4498	92.63	FOA15_RS01880	90.41
	phnC	IAQ67_24270	ABE82_21685	97.80	PPSQR21_043870	93.39	C1A50_4497	93.00	FOA15_RS01875	90.79
	pstB	IAQ67_08060	ABE82_08415	97.98	PPSQR21_016690	90.39	C1A50_1702	90.39	FOA15_RS13995	87.78
	pstA	IAQ67_08055	ABE82_08410	98.33	PPSQR21_016680	92.20	C1A50_1701	92.20	FOA15_RS13990	88.74
	pstC	IAQ67_08050	ABE82_08405	97.32	PPSQR21_016670	93.78	C1A50_1700	93.78	FOA15_RS13985	90.25
	gndA	IAQ67_18670	ABE82_16080	98.01	PPSQR21_031260	94.95	C1A50_3272	94.81	FOA15_RS21710	93.03
	gnd	IAQ67_10415	ABE82_10835	96.53	PPSQR21_021150	90.38	C1A50_2157	90.49	FOA15_RS16350	87.92
	gntK	IAQ67_02200	ABE82_02330	97.48	PPSQR21_004580	90.06	C1A50_0463	90.26	FOA15_RS08185	88.64
	ppc	IAQ67_24795	ABE82_22250	98.10	PPSQR21_044780	91.26	C1A50_4591	91.23	FOA15_RS02325	89.15
	citZ	IAQ67_08085	ABE82_08440	98.11	PPSQR21_016740	93.26	C1A50_1707	93.26	FOA15_RS14020	90.12
	gltB	IAQ67_05685	ABE82_05885	97.89	PPSQR21_011790	92.69	C1A50_1240	92.78	FOA15_RS11615	90.63
	acnA	IAQ67_03535	ABE82_03720	96.78	PPSQR21_007730	91.26	C1A50_0814	91.30	FOA15_RS09550	89.97
	sucC	IAQ67_09630	ABE82_10080	98.88	PPSQR21_019530	94.14	C1A50_2000	93.88	FOA15_RS15585	93.54
	sucD	IAQ67_09635	ABE82_10085	97.53	PPSQR21_019540	92.69	C1A50_2001	92.58	FOA15_RS15590	89.46
	sdhA	IAQ67_22970	ABE82_20370	97.59	PPSQR21_041080	92.21	C1A50_4187	91.92	FOA15_RS00560	90.78
	sdhB	IAQ67_22965	ABE82_20365	98.05	PPSQR21_041070	92.58	C1A50_4186	92.58	FOA15_RS00555	90.62
	fumC	IAQ67_12970	ABE82_13485	96.97	PPSQR21_026040	91.50	C1A50_2718	91.50	FOA15_RS19135	88.11
	mdh	IAQ67_08095	ABE82_08450	97.56	PPSQR21_016760	89.90	C1A50_1709	89.90	FOA15_RS14030	87.22

	pdhA	IAQ67_13430	ABE82_14010	98.88	PPSQR21_026930	94.76	C1A50_2807	94.66	FOA15_RS19570	93.17
	poxB	IAQ67_10510	ABE82_10925	97.57	PPSQR21_021340	90.67	C1A50_2177	90.55	FOA15_RS16430	87.07
	pflA	IAQ67_18230	ABE82_15645	99.21	PPSQR21_030290	92.36	C1A50_3173	93.02	FOA15_RS21240	90.12
	pflB	IAQ67_18235	ABE82_15650	98.59	PPSQR21_030300	92.71	C1A50_3174	92.71	FOA15_RS21245	90.01
	metG	IAQ67_18210	ABE82_15625	97.18	PPSQR21_030250	90.05	C1A50_3169	90.29	FOA15_RS21220	89.85

NA = not available
