# Supplementary material for: Comparative Genomics Insights into a Novel Biocontrol Agent Paenibacillus peoriae Strain ZF390 against Bacterial Soft Rot
Source: Biology (Basel). 2022 Aug 4;11(8):1172. doi: 10.3390/biology11081172 (PMC9404902; doi:10.3390/biology11081172)
Supplement: Supplementary file 1 [file biology-11-01172-s001.zip › Supplementary Table S7.pdf]

Supplementary Table S7 Comparison of genes involved in biofilm formation between strains ZF390, HS311, SQR-21, HY96-2 and PS04.

Trait	Gene name	ZF390	HS311		SQR-21		HY96-2		PS04	
		Locus tag	Locus tag	Identity (%)	Locus tag	Identity (%)	Locus tag	Identity (%)	Locus tag	Identity (%)
Flagellar assembly	*fliE*	IAQ67_09675	ABE82_10125	99.68	PPSQR21_019620	97.78	C1A50_2009	98.10	FOA15_RS15630	95.56
	fliF	IAQ67_09680	ABE82_10130	98.48	PPSQR21_019630	94.13	C1A50_2010	94.32	FOA15_RS15635	91.35
	fliG	IAQ67_09685	ABE82_10135	98.53	PPSQR21_019640	93.51	C1A50_2011	93.51	FOA15_RS15640	92.53
	fliH	IAQ67_09690	ABE82_10140	98.81	PPSQR21_019650	92.59	C1A50_2012	92.47	FOA15_RS15645	90.44
	fliI	IAQ67_09695	ABE82_10145	98.64	PPSQR21_019660	91.44	C1A50_2013	91.06	FOA15_RS15650	89.09
	fliJ	IAQ67_09700	ABE82_10150	99.55	PPSQR21_019670	96.17	C1A50_2014	96.17	FOA15_RS15655	97.07
	fliK	IAQ67_09710	ABE82_10160	97.05	PPSQR21_019690	89.51	C1A50_2016	89.71	FOA15_RS15665	86.79
	fliL	IAQ67_09735	ABE82_10185	99.37	PPSQR21_019740	97.68	C1A50_2021	97.47	FOA15_RS15690	93.88
	fliM	IAQ67_09740	ABE82_10190	99.30	PPSQR21_019750	96.20	C1A50_2022	96.10	FOA15_RS15695	93.89
	fliN	IAQ67_09745	ABE82_10195	98.25	PPSQR21_019760	94.67	C1A50_2023	93.76	FOA15_RS15700	89.47
	fliO	IAQ67_09755	ABE82_10205	99.25	PPSQR21_019780	96.65	C1A50_2025	96.83	FOA15_RS15710	95.32
	fliP	IAQ67_09760	ABE82_10210	98.68	PPSQR21_019790	95.24	C1A50_2026	95.50	FOA15_RS15715	94.44
	fliQ	IAQ67_09765	ABE82_10215	98.89	PPSQR21_019800	96.30	C1A50_2027	96.30	FOA15_RS15720	95.93
	fliR	IAQ67_09770	ABE82_10220	98.35	PPSQR21_019810	92.49	C1A50_2028	93.13	FOA15_RS15725	90.08
	flhA	IAQ67_09780	ABE82_10230	97.89	PPSQR21_019830	92.58	C1A50_2030	92.63	FOA15_RS15735	90.07
	flhB	IAQ67_09775	ABE82_10225	98.83	PPSQR21_019820	93.71	C1A50_2029	94.70	FOA15_RS15730	94.43
	flgB	IAQ67_09665	ABE82_10115	99.26	PPSQR21_019600	96.81	C1A50_2007	96.81	FOA15_RS15620	93.14
	flgC	IAQ67_09670	ABE82_10120	99.56	PPSQR21_019610	97.13	C1A50_2008	97.13	FOA15_RS15625	94.04

flgD	IAQ67_09715	ABE82_10165	97.57	PPSQR21_019700	92.88	C1A50_2017	92.88	FOA15_RS15670	89.33
flgE	IAQ67_09725	ABE82_10175	87.96	PPSQR21_019720	85.26	C1A50_2019	88.24	FOA15_RS15680	85.08
flgF	IAQ67_25870	ABE82_23355	98.31	PPSQR21_046940	91.41	C1A50_4815	91.41	FOA15_RS03425	90.85
flgG	IAQ67_25865	ABE82_23350	98.55	PPSQR21_046930	92.27	C1A50_4814	91.67	FOA15_RS03420	88.15
flgK	NA	ABE82_23205	NA	NA	NA	C1A50_4782	NA	FOA15_RS03275	NA
flgK	IAQ67_25720	NA	NA	PPSQR21_046630	91.71	NA	NA	NA	NA
flgL	NA	ABE82_23200	NA	NA	NA	NA	NA	FOA15_RS03270	NA
flgL	IAQ67_25715	NA	NA	PPSQR21_046620	92.83	NA	NA	NA	NA
fliC	NA	ABE82_23185	NA	NA	NA	C1A50_4778	NA	FOA15_RS03255	NA
fliC	IAQ67_25695	NA	NA	PPSQR21_046580	80.84	NA	NA	NA	NA
fliD	NA	ABE82_23175	NA	NA	NA	NA	NA	FOA15_RS03245	NA
fliD	NA	NA	NA	PPSQR21_046460	NA	NA	NA	NA	NA
fliS	NA	ABE82_23170	NA	NA	NA	NA	NA	FOA15_RS03240	NA
fliS	NA	NA	NA	PPSQR21_046450	NA	NA	NA	NA	NA
motA	IAQ67_00530	ABE82_00515	97.86	PPSQR21_001030	88.55	C1A50_0111	88.55	FOA15_RS06375	85.79
motA	IAQ67_25445	ABE82_22900	98.31	PPSQR21_046080	91.79	C1A50_4728	91.67	FOA15_RS02955	88.33
motB	IAQ67_00525	ABE82_00510	97.46	PPSQR21_001020	91.56	C1A50_0110	91.68	FOA15_RS06370	89.25
motB	IAQ67_25450	ABE82_22905	97.18	PPSQR21_046090	88.98	C1A50_4729	88.97	NA	NA
flgM	IAQ67_25730	ABE82_23215	95.65	PPSQR21_046650	95.29	C1A50_4784	94.93	FOA15_RS03285	90.58
fliY	IAQ67_03860	ABE82_04000	97.90	PPSQR21_008370	90.09	C1A50_0876	89.98	FOA15_RS09820	88.24
fliY	IAQ67_21165	ABE82_18585	96.95	PPSQR21_037380	89.67	C1A50_3827	89.44	FOA15_RS24065	90.85
rpoD	IAQ67_19485	ABE82_16910	98.24	PPSQR21_033900	97.44	C1A50_3447	97.27	FOA15_RS22510	95.24
rpoN	IAQ67_07520	ABE82_12750	97.61	NA	NA	NA	NA	NA	NA

	fliA	IAQ67_09825	ABE82_10275	98.86	PPSQR21_019920	93.54	C1A50_2039	93.66	FOA15_RS15780	92.78
	srfAC	NA	NA	NA	PPSQR21_013120	NA	C1A50_1383	NA	NA	NA
	ituA	NA	ABE82_05825	NA	PPSQR21_011670	NA	C1A50_1227	NA	NA	NA
	ituA	NA	NA	NA	PPSQR21_009720	NA	C1A50_1015	NA	NA	NA
Nonribosomal	*fenC*	IAQ67_12025	ABE82_12415	97.43	NA	NA	NA	NA	NA	NA
peptide structures	*fenB*	IAQ67_24735	ABE82_22155	97.64	PPSQR21_044650	97.37	C1A50_4577	97.79	FOA15_RS17225	77.53
	fenA	NA	ABE82_05815	NA	PPSQR21_011650	NA	C1A50_1225	NA	NA	NA
	fenA	IAQ67_12540	ABE82_12955	99.92	PPSQR21_024990	99.82	C1A50_2567	99.81	NA	NA
	fenA	IAQ67_12545	ABE82_12950	99.93	PPSQR21_025010	99.79	C1A50_2568	99.84	NA	NA
	mcp	IAQ67_02300	ABE82_02430	96.40	PPSQR21_005050	88.23	C1A50_0512	88.11	NA	NA
	mcp	IAQ67_05160	ABE82_05315	97.96	PPSQR21_010680	92.69	C1A50_1117	92.61	FOA15_RS11145	90.09
	mcp	IAQ67_06225	ABE82_06370	92.52	PPSQR21_012710	89.46	C1A50_1339	89.21	FOA15_RS12100	89.52
	mcp	IAQ67_06655	ABE82_06795	97.82	PPSQR21_013630	89.79	C1A50_1435	89.79	FOA15_RS12625	87.42
	mcp	IAQ67_08250	ABE82_08570	96.70	NA	NA	C1A50_1734	90.80	NA	NA
	mcp	IAQ67_09205	ABE82_09550	96.42	PPSQR21_018710	94.31	C1A50_1916	94.31	NA	NA
Bacterial	*mcp*	IAQ67_09235	ABE82_09580	96.97	PPSQR21_018770	89.94	C1A50_1923	90.11	FOA15_RS15200	85.34
chemotaxis	*mcp*	IAQ67_09280	ABE82_09625	97.68	PPSQR21_018860	91.64	C1A50_1933	91.53	FOA15_RS15245	85.31
	mcp	IAQ67_11720	ABE82_12040	96.75	PPSQR21_023520	89.32	C1A50_2424	88.87	FOA15_RS17660	85.04
	mcp	IAQ67_18010	ABE82_15430	97.24	PPSQR21_029860	87.71	C1A50_3126	87.66	FOA15_RS21025	85.30
	mcp	IAQ67_18775	ABE82_16185	96.32	PPSQR21_031570	90.88	C1A50_3304	90.93	FOA15_RS21820	84.03
	mcp	IAQ67_20305	ABE82_17735	97.38	PPSQR21_035680	92.20	C1A50_3645	92.11	FOA15_RS23335	92.54
	mcp	IAQ67_20340	ABE82_17780	97.59	PPSQR21_035780	91.98	C1A50_3658	91.70	FOA15_RS23370	86.61
	mcp	IAQ67_21370	ABE82_18780	91.78	PPSQR21_037690	89.45	C1A50_3863	89.58	NA	NA

mcp	IAQ67_21545	ABE82_18950	96.93	PPSQR21_038040	91.56	C1A50_3898	91.69	FOA15_RS24455	88.31
mcp	IAQ67_21595	ABE82_19000	94.63	PPSQR21_038140	91.49	C1A50_3908	91.08	FOA15_RS24505	86.43
mcp	IAQ67_22455	ABE82_19850	94.34	PPSQR21_040050	89.86	C1A50_4078	89.91	FOA15_RS00090	85.58
mcp	IAQ67_26450	ABE82_23950	93.87	PPSQR21_048070	89.94	C1A50_4935	89.99	FOA15_RS04040	83.98
mcp	NA	ABE82_24725	NA	NA	NA	NA	NA	NA	NA
mcp	IAQ67_08120	ABE82_08475	96.71	PPSQR21_016810	90.04	C1A50_1715	90.04	FOA15_RS14060	86.55
mcp	NA	NA	NA	PPSQR21_036040	NA	C1A50_3685	NA	NA	NA
mcp	NA	NA	NA	PPSQR21_038590	NA	C1A50_3934	NA	NA	NA
mcp	NA	NA	NA	PPSQR21_051220	NA	NA	NA	NA	NA
cheD	IAQ67_09815	ABE82_10265	97.19	PPSQR21_019900	91.16	C1A50_2037	91.16	FOA15_RS15770	91.57
cheR	IAQ67_17590	ABE82_15020	97.85	PPSQR21_029030	94.94	C1A50_3042	95.32	FOA15_RS20610	90.77
cheR	IAQ67_23780	ABE82_21195	96.90	PPSQR21_042930	93.22	C1A50_4394	93.79	NA	NA
cheB	IAQ67_09795	ABE82_10245	97.99	PPSQR21_019860	89.39	C1A50_2033	89.33	FOA15_RS15750	84.12
cheA	IAQ67_09800	ABE82_10250	97.66	PPSQR21_019870	92.70	C1A50_2034	92.61	FOA15_RS15755	90.27
cheW	IAQ67_09805	ABE82_10255	99.57	PPSQR21_019880	93.07	C1A50_2035	93.07	FOA15_RS15760	93.29
cheW	IAQ67_24355	ABE82_21770	96.14	PPSQR21_044060	91.06	C1A50_4518	91.06	FOA15_RS01970	86.96
cheC	IAQ67_09810	ABE82_10260	99.36	PPSQR21_019890	93.46	C1A50_2036	93.30	FOA15_RS15765	89.79
cheX	IAQ67_22920	ABE82_20305	99.13	PPSQR21_040970	96.95	C1A50_4176	96.73	FOA15_RS00500	93.90
cheY	IAQ67_09750	ABE82_10200	99.73	PPSQR21_019770	97.81	C1A50_2024	97.81	FOA15_RS15705	97.50
cheY	IAQ67_10960	ABE82_11350	98.07	PPSQR21_022280	92.11	C1A50_2296	92.11	FOA15_RS16905	90.08
fliG	IAQ67_09685	ABE82_10135	98.53	PPSQR21_019640	93.51	C1A50_2011	93.51	FOA15_RS15640	92.53
fliM	IAQ67_09740	ABE82_10190	99.30	PPSQR21_019750	96.20	C1A50_2022	96.10	FOA15_RS15695	93.89
fliN	IAQ67_09745	ABE82_10195	98.25	PPSQR21_019760	94.67	C1A50_2023	93.76	FOA15_RS15700	89.47

	motA	IAQ67_00530	ABE82_00515	97.86	PPSQR21_001030	88.55	C1A50_0111	88.55	FOA15_RS06375	85.79
	motA	IAQ67_25445	ABE82_22900	98.31	PPSQR21_046080	91.79	C1A50_4728	91.67	FOA15_RS02955	88.33
	motB	IAQ67_00525	ABE82_00510	97.46	PPSQR21_001020	91.56	C1A50_0110	91.68	FOA15_RS06370	89.25
	motB	IAQ67_25450	ABE82_22905	97.18	PPSQR21_046090	88.98	C1A50_4729	88.97	NA	NA
	rbsB	IAQ67_05040	ABE82_05215	98.9	PPSQR21_010490	90.59	C1A50_1098	90.69	FOA15_RS11045	91.59
	rbsB	IAQ67_05325	ABE82_05490	96.73	PPSQR21_010960	89.38	C1A50_1147	89.29	FOA15_RS11265	88.57
	rbsB	IAQ67_13175	ABE82_13755	97.29	PPSQR21_026420	90.33	C1A50_2758	90.76	FOA15_RS19340	86.21
	luxS	IAQ67_02605	ABE82_02735	97.62	PPSQR21_005820	92.42	C1A50_0600	92.42	FOA15_RS08615	91.13
	aphA	IAQ67_02980	ABE82_03245	97.27	PPSQR21_006690	90.35	C1A50_0698	90.71	FOA15_RS09040	87.91
	aphA	IAQ67_17015	ABE82_14410	96.85	PPSQR21_027930	92.50	C1A50_2922	92.50	FOA15_RS19965	89.37
	hfq	IAQ67_16940	ABE82_14335	100.00	PPSQR21_027780	94.31	C1A50_2906	93.50	FOA15_RS19890	91.06
	trpE	IAQ67_17570	ABE82_15000	97.94	PPSQR21_028990	93.36	C1A50_3038	93.29	FOA15_RS20590	90.84
	kdpE	NA	ABE82_13170	NA	NA	NA	NA	NA	NA	NA
	blcC	IAQ67_13135	ABE82_13725	98.68	PPSQR21_026340	92.77	C1A50_2750	93.52	NA	NA
Quorum sensing	*blcC*	NA	ABE82_14430	NA	NA	NA	NA	NA	NA	NA
	blcC	IAQ67_13135	ABE82_13725	98.68	PPSQR21_026350	92.77	C1A50_2750	93.52	NA	NA
	blcC	IAQ67_20745	NA	NA	PPSQR21_034830	99.08	C1A50_3551	99.08	NA	NA
	pel	IAQ67_01205	ABE82_01255	95.37	PPSQR21_002420	89.73	C1A50_0247	89.83	FOA15_RS07185	84.59
	pel	IAQ67_21045	ABE82_18485	96.11	PPSQR21_037180	87.94	C1A50_3806	87.70	FOA15_RS23945	89.60
	fadD	IAQ67_00385	ABE82_00395	96.51	PPSQR21_000780	92.12	C1A50_0084	92.26	FOA15_RS06250	90.05
	crp	IAQ67_27590	ABE82_25150	97.21	PPSQR21_050290	92.74	C1A50_5172	93.11	FOA15_RS05195	87.52
	xagB	IAQ67_25550	ABE82_23005	96.75	PPSQR21_046280	91.75	C1A50_4749	91.79	FOA15_RS03080	87.91
	gmuG	IAQ67_02865	ABE82_03090	95.90	PPSQR21_006420	90.29	C1A50_0666	90.64	NA	NA

gmuG	IAQ67_05775	ABE82_05975	95.63	PPSQR21_011970	91.76	C1A50_1258	91.70	FOA15_RS11710	89.62
gmuG	NA	ABE82_12475	NA	NA	NA	NA	NA	NA	NA
gmuG	IAQ67_19010	ABE82_16445	97.48	PPSQR21_032950	91.38	C1A50_3348	91.38	FOA15_RS22025	85.95
gmuG	NA	ABE82_20505	NA	PPSQR21_025410	NA	C1A50_2657	NA	NA	NA
gmuG	IAQ67_25475	ABE82_22930	96.13	PPSQR21_046130	90.71	C1A50_4734	90.61	FOA15_RS03010	92.10
gmuG	NA	NA	NA	PPSQR21_005920	NA	C1A50_0610	NA	NA	NA
gmuG	IAQ67_02220	NA	NA	PPSQR21_006970	92.73	C1A50_0728	92.42	NA	NA
gmuG	NA	NA	NA	PPSQR21_036480	NA	NA	NA	NA	NA
ydcZ	IAQ67_12155	ABE82_12615	99.77	NA	NA	NA	NA	NA	NA
ydcZ	IAQ67_26040	ABE82_23525	97.76	PPSQR21_047290	90.12	C1A50_4853	88.68	FOA15_RS03610	85.98
ydcZ	IAQ67_26050	ABE82_23535	97.42	PPSQR21_047310	91.28	C1A50_4855	91.31	FOA15_RS03620	86.62
ribA	IAQ67_24380	ABE82_21790	97.78	PPSQR21_044110	92.46	C1A50_4524	92.72	FOA15_RS01990	89.41
ribD	IAQ67_24375	ABE82_21785	96.73	PPSQR21_044100	92.01	C1A50_4523	92.37	FOA15_RS01985	89.22
ydcT	IAQ67_26595	ABE82_24085	98.68	PPSQR21_048360	93.48	C1A50_4964	93.67	FOA15_RS04140	90.27
ydcV	IAQ67_26600	ABE82_24090	98.99	PPSQR21_048370	95.44	C1A50_4965	95.06	FOA15_RS04145	92.52
ydcU	IAQ67_26605	ABE82_24095	97.81	PPSQR21_048380	92.58	C1A50_4966	93.43	FOA15_RS04150	91.73
ydcS	IAQ67_26615	ABE82_24105	96.65	PPSQR21_048400	91.25	C1A50_4969	90.78	FOA15_RS04160	90.69
ydcU	IAQ67_28125	ABE82_25745	97.75	NA	NA	NA	NA	FOA15_RS05625	91.58
ydcS	IAQ67_28130	ABE82_25750	97.01	NA	NA	NA	NA	FOA15_RS05630	84.02
ydcT	IAQ67_28135	ABE82_25755	98.45	NA	NA	NA	NA	FOA15_RS05635	86.24
ydcV	IAQ67_28140	ABE82_25760	98.06	NA	NA	NA	NA	FOA15_RS05640	92.85
agrB	NA	ABE82_07565	NA	NA	NA	NA	NA	NA	NA
agrB	IAQ67_09445	ABE82_09790	97.09	PPSQR21_019140	91.28	C1A50_1961	91.28	NA	NA

agrB	IAQ67_13275	ABE82_13860	93.98	PPSQR21_026620	85.82	C1A50_2775	85.79	NA	NA
oppA	IAQ67_04645	ABE82_04785	97.57	PPSQR21_009960	91.61	C1A50_1038	91.61	FOA15_RS10630	91.06
oppF	IAQ67_19345	ABE82_16775	98.72	PPSQR21_033630	92.97	C1A50_3418	92.76	FOA15_RS22370	90.84
oppC	IAQ67_19350	ABE82_16780	98.42	PPSQR21_033640	95.58	C1A50_3419	95.79	FOA15_RS22375	86.67
oppB	IAQ67_19355	ABE82_16785	98.39	PPSQR21_033650	95.61	C1A50_3420	95.61	FOA15_RS22380	87.78
oppA	IAQ67_19360	ABE82_16790	98.00	PPSQR21_033660	93.24	C1A50_3421	93.18	FOA15_RS22385	91.90
oppD	IAQ67_21050	ABE82_18490	98.69	PPSQR21_037190	95.32	C1A50_3807	95.69	FOA15_RS23950	92.03
ddpD	IAQ67_01880	ABE82_02025	97.09	PPSQR21_003960	91.47	C1A50_0403	91.28	NA	NA
ddpD	IAQ67_10760	ABE82_11145	97.87	PPSQR21_021830	91.36	C1A50_2229	91.46	FOA15_RS16630	89.33
ddpD	IAQ67_12885	ABE82_13400	97.01	PPSQR21_025870	83.87	C1A50_2701	83.63	FOA15_RS19055	81.99
ddpD	IAQ67_13235	ABE82_13820	95.08	NA	NA	NA	NA	NA	NA
ddpD	NA	ABE82_14700	NA	NA	NA	NA	NA	NA	NA
ddpD	IAQ67_20395	ABE82_17885	96.26	NA	NA	NA	NA	FOA15_RS23480	91.41
ddpD	IAQ67_27435	ABE82_24990	96.94	PPSQR21_050010	84.93	C1A50_5141	84.36	FOA15_RS05070	83.19
ddpD	IAQ67_28195	ABE82_25815	96.85	PPSQR21_051510	92.89	C1A50_5306	92.89	FOA15_RS05745	91.06
ddpF	IAQ67_01885	ABE82_02030	96.76	PPSQR21_003970	89.22	C1A50_0404	89.66	NA	-
ddpF	IAQ67_10765	ABE82_11150	98.25	PPSQR21_021840	91.33	C1A50_2230	91.43	FOA15_RS16635	88.21
ddpF	IAQ67_28190	ABE82_25810	97.41	PPSQR21_051500	90.04	C1A50_5305	90.15	FOA15_RS05740	87.53
yidC	IAQ67_11330	ABE82_11640	98.80	PPSQR21_022780	91.85	C1A50_2349	91.73	FOA15_RS17300	89.45
yidC	IAQ67_28300	ABE82_25930	99.31	PPSQR21_051720	92.3	C1A50_5327	92.41	FOA15_RS05855	91.61
srfAC	NA	NA	NA	PPSQR21_013120	NA	C1A50_1383	NA	NA	NA
secG	IAQ67_01010	ABE82_00985	98.29	PPSQR21_001930	95.73	C1A50_0201	95.30	FOA15_RS06820	94.02
ftsY	IAQ67_09535	ABE82_09875	99.21	PPSQR21_019320	91.53	C1A50_1978	91.63	FOA15_RS15490	89.23

ffh	IAQ67_09550	ABE82_09890	98.69	PPSQR21_019350	93.97	C1A50_1981	93.90	FOA15_RS15505	91.43
yidC	IAQ67_11330	ABE82_11640	98.80	PPSQR21_022780	91.85	C1A50_2349	91.73	FOA15_RS17300	89.45
yajC	IAQ67_21750	ABE82_19150	98.21	PPSQR21_038690	94.63	C1A50_3944	94.63	FOA15_RS24835	92.81
secY	IAQ67_24990	ABE82_22445	99.08	PPSQR21_045160	96.54	C1A50_4632	96.54	FOA15_RS02515	94.07
secE	IAQ67_25165	ABE82_22615	99.48	PPSQR21_045510	98.44	C1A50_4670	98.44	FOA15_RS02690	97.40
secA	IAQ67_25610	ABE82_23065	97.84	PPSQR21_046400	92.57	C1A50_4762	92.62	FOA15_RS03140	91.88
yidC	IAQ67_28300	ABE82_25930	99.31	PPSQR21_051720	92.30	C1A50_5327	92.41	FOA15_RS05855	91.61
degU	IAQ67_25760	ABE82_23245	97.93	PPSQR21_046720	92.95	C1A50_4790	93.08	FOA15_RS03315	92.67
spo0F	IAQ67_00715	ABE82_00700	99.73	PPSQR21_001350	95.66	C1A50_0143	95.66	FOA15_RS06530	92.68
spo0B	IAQ67_21895	ABE82_19295	98.51	PPSQR21_038980	93.22	C1A50_3973	92.95	FOA15_RS24980	91.06
spo0A	IAQ67_18025	ABE82_15440	97.64	PPSQR21_029880	93.28	C1A50_3129	93.53	FOA15_RS21040	93.53
nisB	NA	ABE82_08710	NA	NA	NA	NA	NA	NA	NA
nisB	IAQ67_07495	NA	NA	PPSQR21_015860	84.15	C1A50_1611	84.28	NA	NA
nisB	NA	NA	NA	PPSQR21_045960	NA	C1A50_4716	NA	NA	NA
nisB	NA	NA	NA	PPSQR21_048920	NA	NA	NA	NA	NA
nisC	NA	ABE82_08720	NA	NA	NA	NA	NA	NA	NA
nisC	IAQ67_07475	NA	NA	PPSQR21_015820	83.11	C1A50_1608	83.11	NA	NA
nisC	IAQ67_07485	NA	NA	PPSQR21_015840	83.70	C1A50_1609	83.70	NA	NA
nisC	NA	NA	NA	PPSQR21_045950	NA	C1A50_4715	NA	NA	NA
nisC	NA	NA	NA	PPSQR21_048900	NA	C1A50_5022	NA	NA	NA
nisT	NA	ABE82_08715	NA	NA	NA	NA	NA	NA	NA
nisT	IAQ67_07500	NA	NA	PPSQR21_015870	89.86	C1A50_1612	90.12	NA	NA
nisT	NA	NA	NA	PPSQR21_048910	NA	C1A50_5023	NA	NA	NA

	nisK	IAQ67_12835	NA	NA	PPSQR21_025740	90.64	C1A50_2687	90.35	NA	NA
	nisR	IAQ67_12830	NA	NA	PPSQR21_025730	92.41	C1A50_2686	92.41	FOA15_RS18980	88.33
	nisF	IAQ67_12815	NA	NA	PPSQR21_025700	93.64	C1A50_2683	93.50	FOA15_RS18965	85.29
	nisE	IAQ67_12820	NA	NA	PPSQR21_025710	88.66	C1A50_2684	88.11	FOA15_RS18970	81.06
	nisG	IAQ67_12825	NA	NA	PPSQR21_025720	86.96	C1A50_2685	86.96	NA	NA

NA = not available
