# Supplementary material for: Comparative Genomics Insights into a Novel Biocontrol Agent Paenibacillus peoriae Strain ZF390 against Bacterial Soft Rot
Source: Biology (Basel). 2022 Aug 4;11(8):1172. doi: 10.3390/biology11081172 (PMC9404902; doi:10.3390/biology11081172)
Supplement: Supplementary file 1 [file biology-11-01172-s001.zip › Supplementary Table S8.pdf]

Supplementary Table S8 Comparison of genes involved in synthesis of resistance inducers between strains ZF390, HS311, SQR-21, HY96-2 and PS04.

Trait	Gene name	ZF390	HS311		SQR-21		HY96-2		PS04	
		Locus tag	Locus tag	Identity (%)	Locus tag	Identity (%)	Locus tag	Identity (%)	Locus tag	Identity (%)
2, 3-Butanediol	*budA*	IAQ67_10485	ABE82_10900	98.66	PPSQR21_021290	91.3	C1A50_2172	91.57	FOA15_RS16405	87.38
	ilvN	IAQ67_07060	ABE82_07220	99.79	PPSQR21_014480	92.39	C1A50_1525	92.18	NA	NA
	alsD	IAQ67_10485	ABE82_10900	98.66	PPSQR21_021290	91.3	C1A50_2172	91.57	FOA15_RS16405	87.38
Methanethiol	*metH*	IAQ67_13580	ABE82_14160	98.20	PPSQR21_027240	92.36	C1A50_2842	92.24	FOA15_RS19725	89.57
	metE	IAQ67_26535	ABE82_24030	95.73	PPSQR21_048240	88.79	C1A50_4952	88.72	FOA15_RS04100	88.14
	mtnE	IAQ67_20625	NA	NA	NA	NA	C1A50_3727	NA	NA	NA
	mmuM	IAQ67_27500	ABE82_25055	96.1	PPSQR21_050120	87.58	C1A50_5155	87.26	FOA15_RS05125	87.87
Isoprene	*idi*	IAQ67_26035	ABE82_23520	94.82	PPSQR21_047280	86.74	C1A50_4852	87.01	FOA15_RS03605	85.74
	lytB	IAQ67_07305	ABE82_07770	98.01	PPSQR21_015510	92.58	C1A50_1576	92.58	FOA15_RS13505	86.51
	gcpE	IAQ67_22060	ABE82_19460	95.89	PPSQR21_039310	90.88	C1A50_4006	90.88	FOA15_RS25145	89.18
	ispF	IAQ67_25210	ABE82_22655	98.74	PPSQR21_045590	89.92	C1A50_4678	89.71	FOA15_RS02735	91.81
	ispE	IAQ67_00170	ABE82_00175	96.84	PPSQR21_000340	91.70	C1A50_0038	91.70	NA	NA

NA = not available
